# Evaluation of the Beckman Coulter DxC 700 AU chemistry analyzer

**DOI:** 10.1016/j.plabm.2019.e00148

**Published:** 2019-11-20

**Authors:** V.J. Bush, C. Smola, P. Schmitt

**Affiliations:** Bassett Medical Center, Cooperstown, NY, USA

**Keywords:** Performance evaluation, Precision, Correlation, Chemistry analyzer

## Abstract

**Objectives:**

We evaluated and defined the analytical performance of the Beckman Coulter DxC 700 AU analyzer compared to the Siemens Dimension Vista 500 analyzer.

**Design:**

and Methods: Performance characteristics included intra and inter-run precision, linearity/analytical measurement range, method correlation, and reference range verification. A total of 53 assays including 11 critical care, 19 general chemistries, 11 proteins, 10 urines, and 2 CSF analytes were tested. We also evaluated similarities and differences in assay methodologies between the 2 systems.

**Results:**

The DxC 700 AU demonstrated excellent precision, comparable analytical measurement ranges and strong method correlation with the Dimension Vista 500 for most serum/plasma assays. 95% of the intra-run and 95% of the inter-run precision QC levels showed <3.0%CV and <6.0%CV, respectively. None of the deviations were clinically significant. The AMRs for all analytes except 5 met the manufacturer’s stated range. ALP, Lactate, U-glucose and CSF-glucose all recovered above the stated upper limit range, while prealbumin showed a smaller range. All analytes, except 14, showed slopes between 0.9 and 1.1 and/or biases <10%. Only ammonia, ferritin and lipase required significant reference range changes. The urine and CSF assays correlated very well with no adjustments in reference ranges required.

**Conclusions:**

The analytical performance of the DxC 700 AU analyzer was acceptable with only a small number of analytes requiring significant reference range changes.

## Highlights

• Analytical performance of the DxC 700 AU chemistry analyzer is presented.
• Many assays on the DxC 700 AU chemistry analyzer exhibited wider analytical measurement ranges.
• The DxC 700 AU chemistry assays demonstrated excellent inter- and intra-run precision.
• Strong patient correlations were found between the DxC 700 AU and the Siemens Vista 500 analyzers.
• Limited numbers of assays required reference range adjustments.

## 1 Introduction

The DxC 700 AU chemistry analyzer is the latest innovation in a line of chemistry systems for the mid-to high-volume laboratory. The DxC 700 AU is a new combination of hardware and software features from the DxC and AU series of chemistry analyzers. Bassett Medical Center (BMC) in Cooperstown, New York is one of the first laboratories in the United States to adopt the new Beckman Coulter DxC AU 700 instruments, along with Beckman Coulter DxI immunoassay analyzers and Power Express automation lines.

The BMC lab had been using Siemens Dimension Vista 500 and Centaur XP instruments connected by a StreamLab automation line. This equipment ranged in age from 5 years for the Centaur XP, 7 years for the StreamLab, to 9 years for Vista. Due to multiple instrument issues and downtimes for all pieces of equipment, replacements were overdue. The BMC laboratory is the core laboratory for a network of 5 smaller hospital labs and two physician office out-patient labs. The BMC laboratory also provides testing for 35 health centers and 14 school-based clinics. The annual network chemistry test volumes are approximately 1.2 ​M. In efforts to centralize laboratory services, adapt to increasing test volumes, reduce redundancy in non-urgent testing and to save costs, the network laboratories transitioned to a rapid response test menu specific to the needs of their patient populations, primarily by segregating non-acute care and emergency services. Therefore, all non-urgent testing for the network was transitioned to the BMC laboratory. The BMC laboratory anticipated a 17% increase in test volumes by centralizing routine chemistry work. All of the subsidiary laboratories also transitioned to smaller Beckman Coulter equipment (Beckman Coulter AU480 and Access 2 analyzers), to standardize methodologies. This enabled the network to share reagents as all reagent packs for each analyzer are transferrable among sites. This was not possible with our prior systems. Additionally, the use of common assay methods and analyzers across sites allow the use of common reference ranges, quality control programs, inventory control and minimize staff training of shared technologists across sites. Our rural central New York location has made it difficult to recruit qualified licensed technical staff. Not uncommon to our network health system, we are experiencing staff shortages and facing several retirements of experienced technologists. The purpose of moving to the Beckman Coulter automated system was to meet the challenges of staffing and to reduce the high cost of supporting full-service laboratories throughout the network.

BMC performed an extensive validation of the new Beckman Coulter analyzers across the network to adequately prepare the Bassett Healthcare Network system for future workload changes and staffing shortages. We present validation data of the DxC 700 AU chemistry analyzers compared to the Siemens Dimension Vista 500 analyzers. This study is unique because it is the first full validation of the DxC 700 AU with its combined DxC and AU technologies against a known prior analyzer system. We present the analytical performance of the new DxC AU 700 analyzer for 53 analytes (11 critical care, 19 general chemistries, 11 proteins, 10 urines, and 2 CSF assays). Additional assays (i.e. drugs) were evaluated, though these will not be presented in this article.

## 2 Materials and methods

Two Beckman Coulter DxC 700 AU analyzers (Brea, CA USA) were validated against the current Siemens Dimension Vista 500 analyzers (Tarrytown, NY USA). The validation studies included: linearity/analytical measurement range (AMR); intra-run and inter-run precision; method comparison; and reference range verification. Sigma metrics were also evaluated for selected assays. All analyzers were operated according to the manufacturer’s and the laboratory’s standard procedures. Prior to evaluation testing protocols, the analyzers had passed all system setup, calibration and QC testing.

Statistical analyses for precision, linearity/AMR, and method comparison were performed with EP Evaluator version 11.3.0.23 (Burlington, VT USA).

All studies were performed under exemption criteria established by the Bassett Medical Center IRB.

The 53 assays evaluated for performance are listed in Table 1, and grouped by category.

**Table 1:** DxC 700 AU to siemens dimension vista 500 methodology comparisons.

Analytes	DxC 700 AU Methodology	Siemens Dimension Vista 500 Methodology
Critical Care		
Na	Indirect ISE	Indirect ISE
K	Indirect ISE	Indirect ISE
Cl	Indirect ISE	Indirect ISE
CO2	PEP-C Enzymatic	PEP-C Enzymatic
Albumin^a^	BCG	BCP
BUN/Urea nitrogen	Urease GLDH Enzymatic	Urease GLDH Enzymatic
Calcium^a^	Arsenazo III, Bichromatic	Cresolphthalein Complexone, Bichromatic
Creatinine^a^	Kinetic Modified Jaffe, IDMS Traceable	Kinetic Creatininase/Peroxide, IDMS Traceable
Glucose	Hexokinase-UV/NAD-NADH	Hexokinase-UV/NAD-NADH
Phosphorus	Phosphomolybdate-UV	Phosphomolybdate-UV
Total Protein	Biuret	Biuret

General Chemistry		
ALP	p-NPP/AMP IFCC	p-NPP/AMP IFCC
ALT	L-Alanine-KG/NADH to NAD IFCC	L-Alanine-KG/NADH to NAD IFCC
Ammonia	Glutamate Dehydrogenase	Glutamate Dehydrogenase
Amylase	CNPG3 Enzymatic	CNPG3 Enzymatic IFCC
AST	L-Aspartate KG/NADH to NAD, UV	L-Aspartate KG/NADH to NAD, UV
Cholesterol	Cholesterol Esterase/Peroxidase, Bichromatic	Cholesterol Esterase/Cholesterol Oxidase, Polychromatic
CK	CP/ADP/NADP to NADPH IFCC	CP/ADP/NADP to NADPH IFCC
DBIL^a^	Diazotized DPD	Diazotized Sulfanilic Acid
GGT	GCNA IFCC	GCNA IFCC
HDL-Chol	Accelerator Selective Detergent, Bichromatic	Liquid Selective Detergent, Bichromatic
Iron^a^	TPTZ Colorimetric	Ferene without Prior Protein Removal
Lactate^a^	Lactate to Pyruvate/H2O2/Peroxidase	Lactate to Pyruvate/NAD to NADH
LD	L-Lactate/NAD to NADH IFCC	L-Lactate/NAD to NADH IFCC
LDL-Chol	Cholesterol Esterase/Peroxidase, Bichromatic	Cholesterol Esterase/Peroxidase, Bichromatic
Lipase^a^	Imamura Di-Glyceride	Methyl Resorufin Ester Substrate
Magnesium	Colorimetric Xylidyl Blue	Colorimetric Methylthymol Blue
TBIL^a^	Diazotized DPD	Diazotized Sulfanilic Acid
Triglyceride	Enzymatic GPO-Trinder	Enzymatic GPO-Trinder
Uric Acid	Uricase	Uricase

Proteins		
Complement C3^a^	Immunoturbidimetric	Nephelometric
Complement C4^a^	Immunoturbidimetric	Nephelometric
CRP^a^	Immunoturbidimetric	Nephelometric
CRP-hs^a^	Latex Particle Immunoturbidimetric	Nephelometric
Ferritin^a^	Latex Particle Immunoturbidimetric	Homogeneous, Sandwich Chemiluminescent Immunoassay
IgA^a^	Immunoturbidimetric	Nephelometric
IgG^a^	Immunoturbidimetric	Nephelometric
IgM^a^	Immunoturbidimetric	Nephelometric
Prealbumin^a^	Immunoturbidimetric	Nephelometric
RF^a^	Immunoturbidimetric	Nephelometric
Transferrin^a^	Immunoturbidimetric	Nephelometric

Urines		
U-Albumin^a^	Immunoturbidimetric	Nephelometric
U-BUN	Urease GLDH Enzymatic	Urease with GLDH
U-Calcium^a^	Arsenazo III, Bichromatic	Cresolphthalein Complexone, Bichromatic
U-Chloride	Indirect ISE	Indirect ISE
U-Creatinine^a^	Kinetic Modified Jaffe, IDMS Traceable	Kinetic Creatininase/Peroxide, IDMS Traceable
U-Glucose	Hexokinase-UV/NAD	Hexokinase-UV/NAD
U-Phosphorus	Phosphomolybdate-UV	Phosphomolybdate-UV
U-Potassium	Indirect ISE	Indirect ISE
U-Sodium	Indirect ISE	Indirect ISE
U-Total Protein	Pyrogallol Red	Pyrogallol Red

CSF		
CSF-Glucose	Hexokinase-UV/NAD	Hexokinase-UV/NAD
CSF-Total Protein	Pyrogallol Red	Pyrogallol Red

This table includes a list of methodologies for each assay tested on each platform. The methodologies were taken from the respective manufacturer’s assay IFU.

a Footnoted assays indicate differing methodologies.

### 2.1 Linearity

Linearity and verification of the lower limit of quantification were performed for each test according to CLSI EP06 [1]. Verichem Laboratories, Inc (Providence, RI USA) standard kits were used for establishing the linearity for most assays. Beckman Coulter SPM calibrators were used for the proteins and urine albumin. Each level for each material was analyzed in triplicate for verifying the analytical measurement range (AMR). Both accuracy and precision of each level had systematic allowable error (SEa) and random allowable error (REa) criteria set, respectively. The lower limit of quantification was determined by diluting either a low calibrator or patient sample to the manufacturer’s target and analyzed in 5 replicates.

### 2.2 Precision

Precision studies were performed using two levels of quality control for intra-run precision, two levels for most inter-run precision assays with three levels for lipids, bilirubins, and proteins. Quality control materials (QC) were obtained from Bio-Rad (Irvine, CA USA). Intra-run precision was evaluated using 20 replicates of each level of QC for each analyte. Due to resource limitations, a complete inter-run experiment as described by CLSI EP05 [2] was not performed. We evaluated one replicate per run, one run per day for most assays except creatinine which had 3 runs per day, for ≥29 days. Intra-run and inter-run precision acceptance limits were set at 25% of allowable CLIA TEa limits. The %CVs were also examined and expected to be no greater than 6%. All results were evaluated for clinical acceptability. Inter-run precision data analysis (mean, SD, %CV) was completed in the laboratory’s EPIC Beaker LIS (Madison, WI USA).

### 2.3 Correlation

Correlation studies were performed according to CLSI EP09 [3], except samples were analyzed in singlet instead of duplicate. Fresh or frozen/thawed patient specimens were used for evaluation of the method correlation. Analysis of specimens between analyzers was completed within 2 ​h. At least 40 patient serum/plasma, CSF and urine specimens for each specimen type were included for each analyte. Deming regression analysis was performed to calculate the slope, intercept, correlation coefficient (R), mean X and Y, and Standard Error of Estimate (SEE). A slope of 0.90–1.10 was considered acceptable. Where values fell outside this expected range, biases were evaluated using difference plots and compared against clinical acceptability.

### 2.4 Reference ranges

Reference ranges were verified using specimens collected from volunteers with selection/exclusion criteria according to CLSI EP28-A3c [4].

Approximately 70 male and female volunteers were recruited for the study. Ages ranged from 18 to 70 years, equally distributed between males and females. SST (Ref # 367983) and PST (Ref # 367960) BD Vacutainer™ tubes (Franklin Lakes, NJ USA) were collected from each donor. All specimens were processed according to the laboratory’s standard procedure. SST tubes were allowed to clot for 30 ​min, specimens were centrifuged at 1500×*g* for 10 ​min within 2 ​h of collection and then aliquoted. The aliquots were frozen at −20 ​°C until analysis could be performed.

Data were reviewed for outliers according to CLSI EP28. Briefly, this entails calculating the upper and lower quartile boundaries and removing extreme values outside these boundaries which do not appear to represent reference range values. A small number of significant outliers were removed from the data set leaving a total of 67 male and female donors. Where significant methodology differences existed, the manufacturer’s reference ranges were adopted based upon the verification data. We maintained national guidelines for clinical decision limits in place of reference ranges where they were already in-use. These included: the NCEP practice guidelines for lipids [5], ADA practice guidelines for glucose [6], KDIGO practice guidelines for urine albumin [7], and ACC/AHA practice guidelines for CRP and hsCRP [8].

### 2.5 Sigma metrics

Sigma metric analysis for representative analytes was calculated according to Westgard [9] using the following equation where TEa is the total allowable error and CV is the coefficient of variation. TEa sources are from CLIA 88′ or Physiological Variation (PV) with source listed [14,15]. Bias values were derived from external proficiency testing peer method performance and CV (%) was obtained from inter-run precision data.

Sigma = (TEa-Bias_observed_)/CV_observed_ with all terms expressed as %

## 3 Results and discussion

Two DxC 700 AU analyzers were evaluated for all assays. The data from the two units were essentially identical with the detailed data from one unit as presented below.

The data presented represents 53 assays including 11 critical care, 19 general chemistries, 11 proteins, 10 urines, and 2 CSF analytes. Complete evaluation of all analytes included: AMR verification, precision (intra-run and inter-run), method comparison and reference range verification. Selected analytes were evaluated for Sigma metrics performance.

The assay methodologies are listed in Table 1 and compared with those of the prior Dimension Vista 500 system. The asterisked assays show 42% of assays with differing methodologies between the 2 analyzer systems.

### 3.1 Linearity/analytical measurement range (AMR)

The acceptance criteria for accuracy and linearity were based on the SEa. REa was used for the acceptance criteria for the replicates at each level. Where the manufacturer stated lower limits down to zero or the linearity material did not reach the lower limit claim, the laboratory chose values verified by the lower limit verification studies. The AMR of each assay was found to be linear within the manufacturer’s specified ranges.

In comparing the Beckman Coulter AMR to the Siemens AMR for each analyte, we found only 9% of assays were similar. The AMR with the Beckman Coulter reagents showed 54% with wider AMRs and 36% with narrower AMRs compared to Siemens. The narrower ranges were usually due to a lower upper limit of detection. All materials recovered within ±20% of the manufacturer’s claim, except 5 methods as shown in Table 2. ALP, Lactate, U-glucose and CSF-glucose all recovered above the stated upper limit. Prealbumin fell below the manufacturer’s claimed upper limit, but was still 20 ​mg/dL higher than the Dimension Vista 500 AMR. This verification allowed us to use the manufacturer’s values to set our method upper limits. For prealbumin, we set the method upper measurement limit at 60 ​mg/dL to match our observed study.

**Table 2:** Analytes with AMR exceptions.

Assay	Units	Manufacter’s Upper Limit Claim	Observed Upper Limit	Recovery
ALP	U/L	1500	1600	107%
Lactate	mmol/L	10	16.5	165%
Prealbumin	mg/dL	80	60	75%
U-Glucose	mg/dL	700	970	138%
CSF-Glucose	mg/dL	800	1000	125%

Analytes with deviations ​> ​±20% above or below the manufacturer’s claimed upper limit of the analytical measurement range. Values recovered from Verichem linearity material for ALP, Lactate, U-Glu and CSF-Glu. Beckman Coulter control material was used for Prealbumin.

Complete Linearity/AMR data are presented in Supplemental Data Table 3.

**Table 3:** Example method correlation data between DxC 700 AU and vista 500.

**Albumin**							


	Slope	Intercept	R	S.E.E.	Mean X	Mean Y	N
	0.825	0.77	0.9916	0.11	3.06	3.3	45

**GGT**							


	Slope	Intercept	R	S.E.E.	Mean X	Mean Y	N
	0.720	1.2	0.9999	2.8	206.3	149.8	40

**CRP**							


	Slope	Intercept	R	S.E.E.	Mean X	Mean Y	N
	1.006	0.7591	0.9947	1.3149	13.1164	13.95	36

Method correlation data was generated per CLSI EP9 and analyzed with EP Evaluator software. 53 Analytes were evaluated including 11 critical care, 19 general chemistries, 11 proteins, 10 urines, and 2 CSF. Three example analytes are shown in this table. All 53 are shown in Supplemental Data Table 4.

### 3.2 Precision

Intra-run precision data included two levels of quality control for each analyte. 25% of the CLIA TEa SD for each method was used as the precision verification goal [10]. The SD’s and CV’s for all analytes are shown in Supplemental Data Table 1. Five assay levels out of 106 evaluated demonstrated intra-run CVs >3.0%, ranging from 3.2 to 4.4% CV. All remaining 101 evaluations were <3.0% CV. All 106 evaluations demonstrated SD’s within the allowable SD test specifications and were clinically acceptable. None of the assays tested exceeded the manufacturer’s defined goals for intra-run precision.

Inter-run precision data shown in Supplemental Data Table 2 included two or three levels of quality control depending upon the assay. Two levels of QC were analyzed for most analytes; three levels of QC were run for lipids, ammonia, direct and total bilirubin and the proteins. We obtained 29–43 days of QC values to determine the inter-run precision for most assays. Creatinine’s more frequent calibration and QC generated significantly more data points, but is also a workflow limitation. The inter-run precision goals were set as 25% of the CLIA TEa SD and by examining the %CV for each assay. Outliers were defined by values exceeding ±3 SD of the laboratory’s preset SD and excluded from the calculations. All levels of QC for all analytes were within the allowable error SD, except for certain levels of iron, ferritin, and CO2 which were subsequently deemed clinically acceptable.

We expected that the Inter-run %CV would be no greater than 6%. The highest CV was 9.8% for low level ferritin, with a SD of 2.6 ​at a mean value of 26.5 ​ng/mL. The two higher ferritin levels had CVs less than 6%. The next highest CVs were 8.6% and 7.2% for low and high level CO2, 6.6% for low level prealbumin, and 6.1% for low level lipase and for mid-level ammonia. We determined that these CVs would unlikely impact patient care. Overall, 96% of 121 Inter-run Precision CV’s were less than 6%.

### 3.3 Method correlation

We compared 53 assays between the Siemens Dimension Vista 500 and Beckman Coulter DxC 700 AU instruments. Example method correlations are shown in Table 3, while the complete data set for all 53 assays is shown in Supplemental Data Table 4 Method Correlation Data. Specimens were collected to obtain a distribution of results across respective measurement ranges. Seventy-four percent of all analytes measured demonstrated slopes of 0.9–1.1. Fifteen assays exhibited slopes <0.9 or >1.1 where 5 of these 15 assays differed in methodology (Table 4). The R can be used in a limited fashion to detect problems with precision, but can be unduly influenced by extremely high or low values. In these cases, standard error of the estimate (SEE) and difference plots have more value in interpreting the data. SEE estimates the distribution of points around the line that is independent of the slope of the line, while bias plots can provide the magnitude of bias for each matched subject’s data. Eighty one percent of the assays showed average biases within ±10%.

**Table 4:** Method correlation exceptions.

Analyte	Slope	Intercept	R	% Bias
General Chemistry				
Albumin^a^	0.825	0.8	0.9916	7.7
ALP	0.883	−4.6	0.999	−13.7
ALT	0.818	−2.8	0.9971	−20.4
Ammonia	1.131	−0.5	0.9964	12.8
Amylase	0.916	−14.5	0.9962	−15.0
AST	0.738	2.1	0.9980	−24.9
CK	0.855	11.2	0.9996	−12.7
DBIL^a^	0.835	0.0	0.9967	−17.1
GGT	0.720	1.2	0.9999	−27.4
LD	0.888	−5.9	0.9963	−13.1
Lipase^a^	0.265	−13.7	0.9765	−78.4
Proteins				
C4^a^	1.423	−4.9	0.9892	24.2
RF^a^	0.693	16.7	0.9413	−9.8
Urine				
U -Chloride	0.887	5.3	0.9949	−5.9
U-Sodium	1.118	−2.3	0.9924	8.7

Analytes with method comparison slopes <0.9 or >1.1 and/or % Biases >10%. % Biases calculated from external proficiency peer data.

^a^ Footnoted assays indicate differing methodologies.

As was shown in the methodology comparison table (Table 1), 58% of the methods were similar between vendors. It cannot be determined by this study design which result may be more accurate, but large biases were not expected based upon similar methodologies (see Table 1) and review of external proficiency testing results. Although the principles of the methods may be similar, the formulations of the reagents or calibration traceability schemes may differ. This may account for some biases that were identified among similar methodologies. Specimen stability may have impacted some correlations as it is well known that ammonia values increase over time and bilirubin will degrade when exposed to light even over short periods of time. Calibration traceability differences may explain the poor correlations noted for the enzymes (AMY, ALP, ALT, AST, CK, GGT, LD). These enzymes, exhibited negative biases >10% but <20% relative to the Dimension Vista 500. The DXC 700 AU enzyme assays are not calibrated, instead rates of reaction are calculated based upon extinction coefficient set points and then verified by testing against standards. The Dimension Vista 500 enzyme assays are 2-point calibrations using specific calibrator material. GGT showed a correlation slope of 0.72 against the Dimension Vista 500, though the intercept (1.2) was very low and R (0.9999) was very high (See Supplemental Data Table 4). This usually indicates a methodology difference and bias. The methods are nominally the same between the 2 systems, but substantive differences must exist in implementation. Despite the biases for ammonia, direct bilirubin and GGT, only small alterations to the reference range were needed. Lipase observed differences were significant (slope ​= ​0.265, intercept ​= ​−13.7) though the correlation was strong (R ​= ​0.9765). This was as predicted by the known nature of the different lipase methodologies and proficiency peer group evaluations. These were large enough to warrant significant reference range changes for lipase.

Differences in albumin values between BCG and BCP dye binding methods for normal subjects are negligible, but this is not true for dialysis patients [11]. Although not specifically tracked during the correlation study, the data set does include dialysis patients. These patient samples were particularly useful in obtaining elevated BUN and creatinine values. We adjusted the serum albumin reference range as specified in the DxC 700 AU instructions for use (IFU) and verified by our reference range study. The switch from BCP to BCG allowed us to remove an LIS calculation for correcting the BCP albumin in the dialysis module of the EMR. Serum albumin values are monitored in dialysis patients as a quality measure that can impact reimbursement of treatment [12]. The national target albumin levels are based upon BCG albumin measurements [13].

Finally, methodology differences exist for the special proteins. The Dimension Vista 500 utilized nephelometric while the DxC 700 AU uses immunoturbidimetric detection methods. These assays are both dependent upon the formation of immune complexes. The specific antibodies used by each vendor may target different epitopes of the protein binding sites thus accounting for some biases. RF demonstrated the poorest regression parameters with a slope of 0.70 and intercept of 16.65. Greater deviations were observed at higher concentrations. RF is a non-specific test that utilizes a titer cut-off for positivity. The cut-off limits for a positive titer were the same for both methods. The biases we observed for RF in the validation study were consistent with biases reported by external proficiency programs.

### 3.4 Reference range verification

We verified reference ranges by testing 67 male and female normal volunteers that met the study inclusion criteria. Both serum and heparin plasma samples were collected from each donor. The laboratory receives a mix of serum and plasma specimens for testing of the same analytes. PST (heparinized) tubes are used primarily for stat testing. Plasma tubes were not analyzed for GGT because the DxC 700 AU IFU advises against the use of heparinized plasma due to the production of turbidity in the reaction. This is a limitation compared to the Vista 500 which allowed heparinized plasma for GGT testing. Plasma total protein values tend to be higher than serum due to the presence of fibrinogen. This study showed no significant differences (≤0.5 ​g/dL) in the matched sample pairs between analyzers. We found no clinically significant differences between serum and plasma for any of the other analytes tested, except potassium. Based upon this data, we maintained separate serum and plasma reference ranges for potassium as shown in Supplemental Data Table 3 Limits.

Glucose, CRP, urine albumin, total cholesterol, HDL-cholesterol, LDL-cholesterol and triglyceride use national risk classification guidelines instead of reference ranges. It was clear from strong correlations, minimal observed biases and the normal donor study that these reference ranges could remain. Likewise, no changes were made to the urine or CSF chemistry analytes. Most other analytes compared very well, but due to slight differences in methodology or reagent formulations we elected to make small adjustments to several reference ranges according to the Beckman Coulter IFU.

A comparison of all the reference ranges used with the Dimension Vista 500 and the DxC 700 AU are shown in Supplemental Data Table 3.

### 3.5 Sigma metrics

**Fig. 1:**
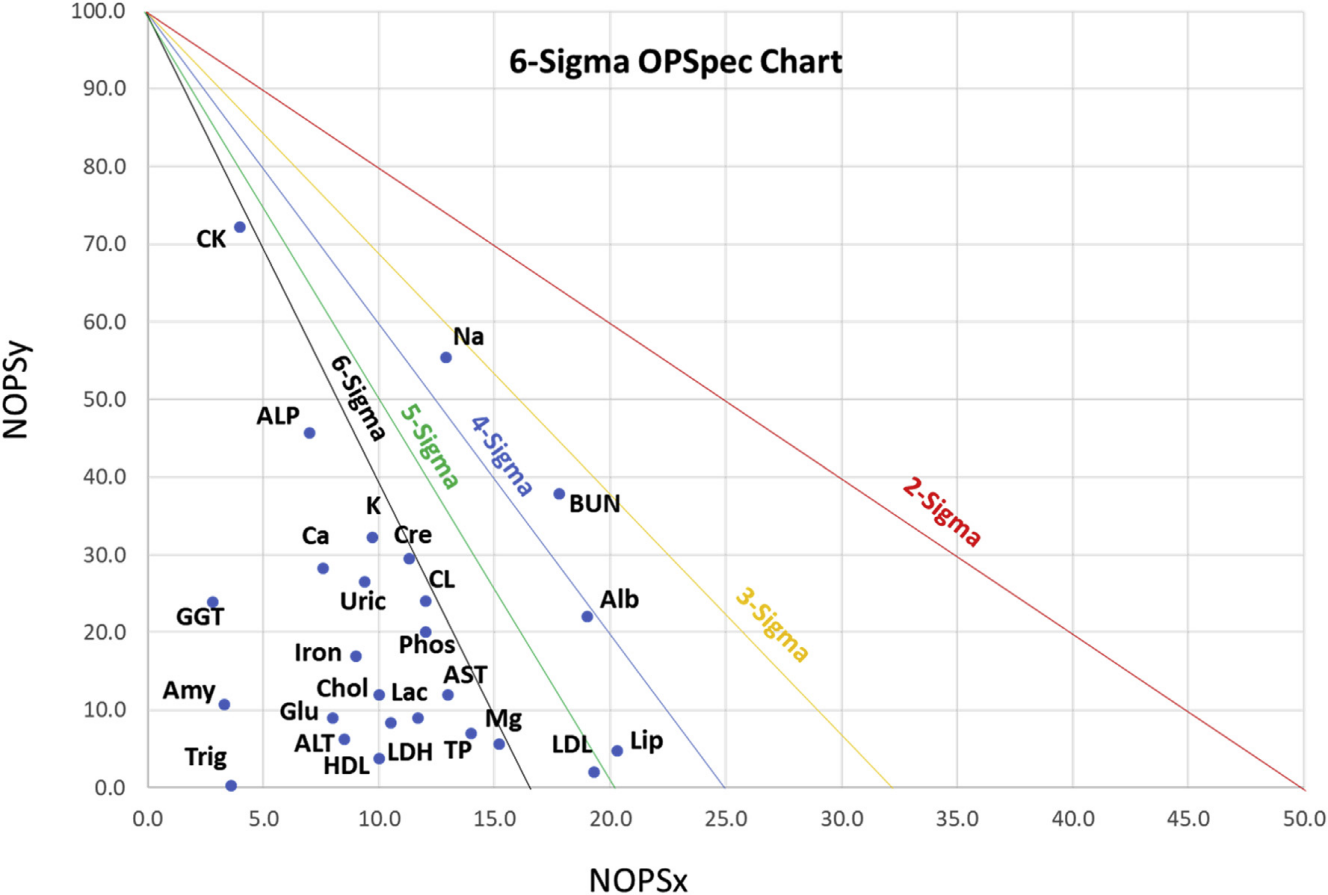
Sigma Metrics of Selected Assays on DxC 700 AU. Sigma values displayed for 26 selected chemistry tests. Refer to Supplemental Data Table 5 for details.

Sigma metrics have become a measure of how well an assay performs within its TEa. These values can assist in defining QC frequency and rules assignments. We analyzed several assays for their sigma performance. The Sigma Metrics of Selected Assays on DxC 700 AU is shown in Fig. 1. Higher sigma values are in the lower left corner. Values are shown in Supplemental Data Table 5. In general, sigma values ​< ​3 are considered poor, values of 4–5 are borderline, while >5–6 are considered good. Of 26 assays evaluated for sigma metric performance, none were <3 sigma, and 22 (85%) were above 5 sigma.

## 4 Limitations

One limitation is that not all of the same patient samples were measured for all analytes although the same samples were assayed on both test platforms. In order to obtain values across the measurement ranges for each analyte, different patient samples were saved for analysis. Another limitation is that some of the specimens were fresh and others had been stored frozen, but this reflects the types of specimens received in the laboratory. Only adults were used for the reference range study. Additionally, we evaluated two DxC 700 AU analyzers, but the data described and presented here are for the performance of one of them. Both analyzers gave very similar performance, and we believe the results to be representative of general performance of the DxC 700 AU analyzer and its assays.

## 5 Conclusions

We have described the validation of the Beckman Coulter DxC 700 AU analyzer and compared it to the Siemens Dimension Vista 500. The DxC 700 AU demonstrated excellent precision, comparable analytical measurement ranges and strong method correlation with the Dimension Vista 500 for most assays. 52% of the assays are similar between platforms. Although we adjusted several reference ranges, due to the strong correlations others may find this unnecessary. Urine and CSF assays correlated very well with no adjustments in reference ranges required.

This evaluation was performed on 2 analyzers and in one laboratory. Both analyzers gave very similar performance, and we believe the results to be representative of general performance of the DxC 700 AU analyzer and its assays.
