# Supplementary material for: Evaluation of the Beckman Coulter DxC 700 AU chemistry analyzer
Source: Pract Lab Med. 2019 Nov 20;18:e00148. doi: 10.1016/j.plabm.2019.e00148 (PMC6909053; doi:10.1016/j.plabm.2019.e00148)
Supplement: Multimedia component 1 [file mmc1.zip › Data-in-brief.docx]

***Data in Brief***

*Version 3.0*

**Article Title**

Supplemental Data for Evaluation of the Beckman Coulter DxC 700 AU Chemistry Analyzer

**Authors**

V.J. Bush^1^, C. Smola^1^, P. Schmitt^1^

**Affiliations**

Bassett Medical Center, Cooperstown, NY

**Corresponding author(s)**

V.J. Bush

valerie.bush@bassett.org

**Abstract**

The supplemental data is related to the article entitled “Evaluation of the Beckman Coulter DxC 700 AU Chemistry Analyzer”. The data provides one lab’s performance with the new Beckman Coulter DxC AU 700 compared to an older Siemen’s Vista 500. Performance characteristics included intra and inter-run precision, linearity/analytical measurement range, method correlation, reference range verification and calculated sigma values. A total of 53 assays including 11 critical care, 19 general chemistries, 11 proteins, 10 urines, and 2 CSF analytes were tested. Herein is a summary of the data for all analytes tested.

**Keywords**

Performance evaluation, precision, linearity, correlation, chemistry analyzer

**Specifications Table**

| **Subject** | Biochemistry, Genetics and Molecular Biology |
| --- | --- |
| **Specific subject area** | Clinical Biochemistry |
| **Type of data** | Tables |
| **How data were acquired** | Beckman Coulter DxC AU 700 and Siemens Vista 500 |
| **Data format** | Analyzed |
| **Parameters for data collection** | Biorad quality control material was used for precision studies; Verichem standards and Beckman Coulter SPM materials were used for linearity studies; patient specimens were used for the correlation and reference range verification studies. |
| **Description of data collection** | The supplemental data tables include performance characteristics including intra and inter-run precision, linearity/analytical measurement range, method correlation, reference range verification for all analytes tested and calculated sigma values for selected analytes. |
| **Data source location** | Bassett Medical Center  Cooperstown, NY  USA  42-41'59'' N, 74-55'48'' W |
| **Data accessibility** | Mendeley doi: 10.17632/drxb6tdc57.1 |
| **Related research article** | V.J. Bush, C. Smola, P. Schmitt  Evaluation of the Beckman Coulter DxC 700 AU Chemistry Analyzer  Practical Laboratory Medicine  DOI |

**Value of the Data**

- The supplemental data illustrate the performance characteristics of the new Beckman Coulter DxC AU 700 analyzer
- The data may benefit other laboratories considering a similar change in chemistry equipment
- The data could be used as a benchmark from one laboratory’s evaluation of the new Beckman Coulter DxC AU 700 analyzer
- The supplemental data tables include the evaluation statistics of all analytes tested

**Data**

Supplemental Data Table 1. Intra-run Precision

Supplemental Data Table 2. Inter-Run Precision

Supplemental Data Table 3. Reference Ranges and AMR Limits

Supplemental Data Table 4. Method Correlation Data

Supplemental Data Table 5. Sigma Values Data Table

**Experimental Design, Materials, and Methods**

Performance characteristics included intra and inter-run precision, linearity/analytical measurement range, method correlation, and reference range verification. Most studies were conducted following CLSI guidelines (refs 2-5). Intra-run precision was evaluated using 20 replicates of each level of QC for each analyte. One replicate per run, one run per day for most assays except creatinine which had 3 runs per day, for ≥29 days. At least 40 patient serum/plasma, CSF and urine specimens for each specimen type were included for each analyte. A total of 53 assays including 11 critical care, 19 general chemistries, 11 proteins, 10 urines, and 2 CSF analytes from patient samples were tested. Statistical analyses for precision, linearity/AMR, and method comparison were performed with EP Evaluator version 11.3.0.23 (Burlington, VT USA).

**Acknowledgments**

We thank E. Austin, S. Horth and S. Szarejko for securing and running samples and P. Joseph for data entry. We also wish to thank Beckman Coulter for support of this study, and Dr. Jack Zakowski for editorial assistance.

**References**

[1] V.J. Bush, C. Smola, P. Schmitt, Evaluation of the Beckman Coulter DxC 700 AU Chemistry Analyzer. Pract Lab Med, submitted July, 2019.

[2] CLSI. Evaluation of the linearity of quantitative measurement procedures: a statistical approach; approved guideline. CLSI document EP06-A. Wayne, PA: Clinical and Laboratory Standards Institute; 2003.

[3] CLSI. Evaluation of precision of quantitative measurement procedures; approved guideline - third edition. CLSI document EP05-A3. Wayne, PA: Clinical and Laboratory Standards Institute; 2014.

[4] CLSI. Measurement procedure comparison and bias estimation using patient samples; approved guideline. CLSI document EP09-A3.Clinical and Laboratory Standards Institute; Wayne, PA: 2013.

[5] CLSI. Defining, establishing and verifying reference intervals in the clinical laboratory; approved guideline. CLSI document EP28-A3c. Clinical and Laboratory Standards Institute; Wayne, PA: 2010.
