# Supplementary material for: Evaluation of the Beckman Coulter DxC 700 AU chemistry analyzer
Source: Pract Lab Med. 2019 Nov 20;18:e00148. doi: 10.1016/j.plabm.2019.e00148 (PMC6909053; doi:10.1016/j.plabm.2019.e00148)
Supplement: Multimedia component 1 [file mmc1.zip › Supplemental Data Table 4. Method Correlation Data.pdf]

**Supplemental Data Table 4.  Method Correlation Data**

Method correlation data was generated per CLSI EP9 and analyzed with EP Evaluator software. All 53 Analytes were evaluated including 11 critical care, 19 general chemistries, 11 proteins, 10 urines, and 2 CSF.

## CRITICAL CARE

### Na

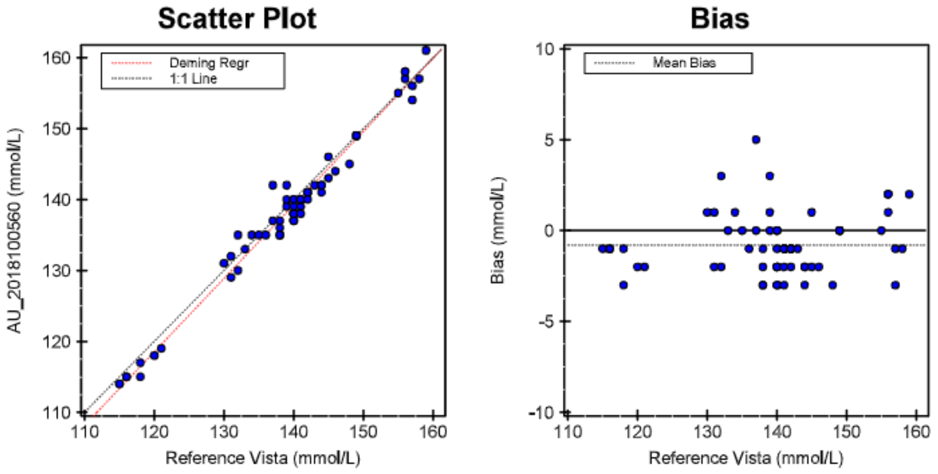


| Slope | Intercept | R | S.E.E. | Mean X | Mean Y | N |
|---|---|---|---|---|---|---|
| 1.035 | -5.6 | 0.9880 | 1.7 | 139.6 | 138.8 | 62 |

### K

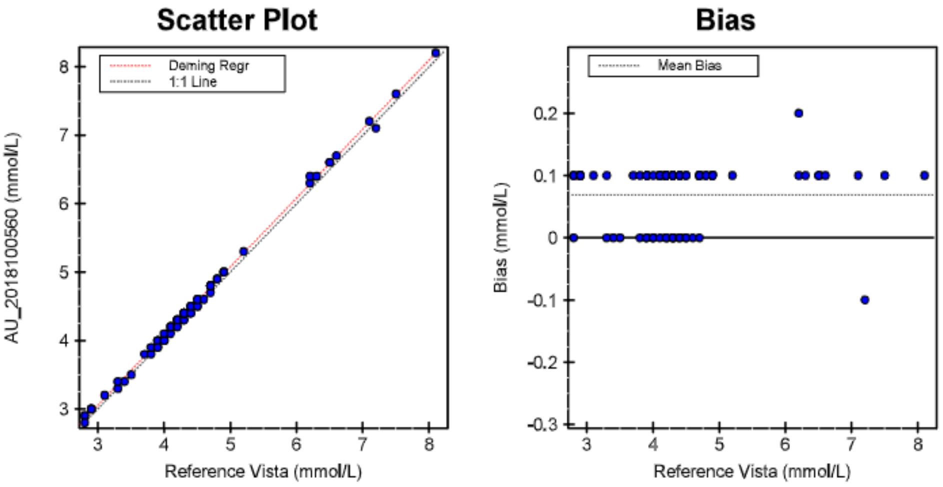


| Slope | Intercept | R | S.E.E. | Mean X | Mean Y | N |
|---|---|---|---|---|---|---|
| 1.007 | 0.04 | 0.9989 | 0.05 | 4.48 | 4.55 | 71 |

### Cl

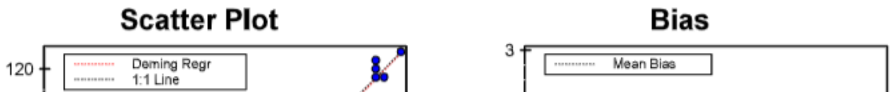

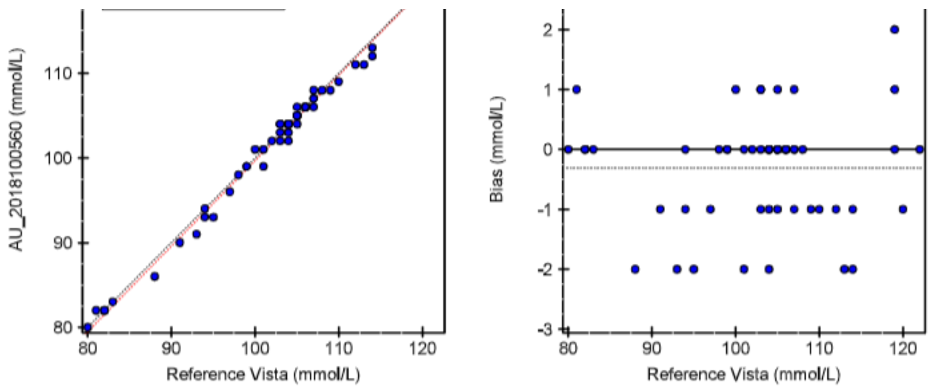


| Slope | Intercept | R | S.E.E. | Mean X | Mean Y | N |
|---|---|---|---|---|---|---|
| 1.009 | -1.2 | 0.9957 | 0.9 | 102.9 | 102.5 | 55 |

**CO2**

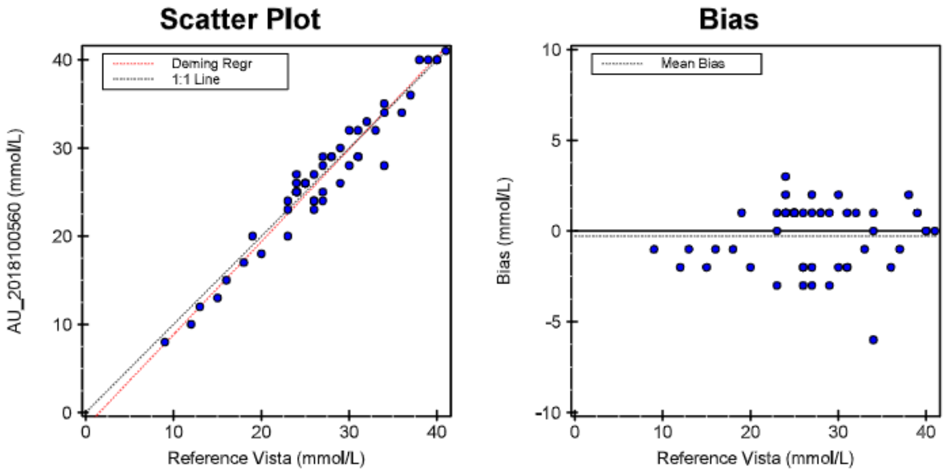


| Slope | Intercept | R | S.E.E. | Mean X | Mean Y | N |
|---|---|---|---|---|---|---|
| 1.050 | -1.6 | 0.9719 | 1.8 | 27.1 | 26.8 | 50 |

**Albumin**

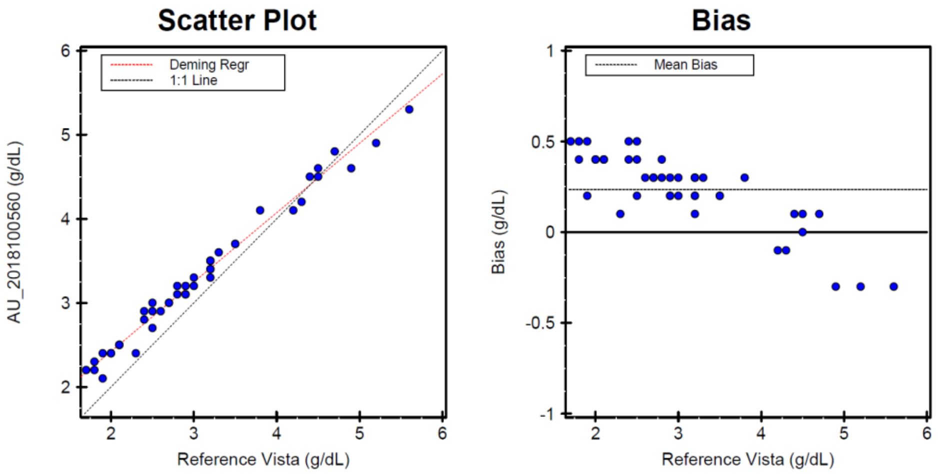


| Slope | Intercept | R | S.E.E. | Mean X | Mean Y | N |
|---|---|---|---|---|---|---|

| | | | | | | |
|---|---|---|---|---|---|---|
| 0.825 | 0.77 | 0.9916 | 0.11 | 3.06 | 3.3 | 45 |

**BUN/Urea Nitrogen**

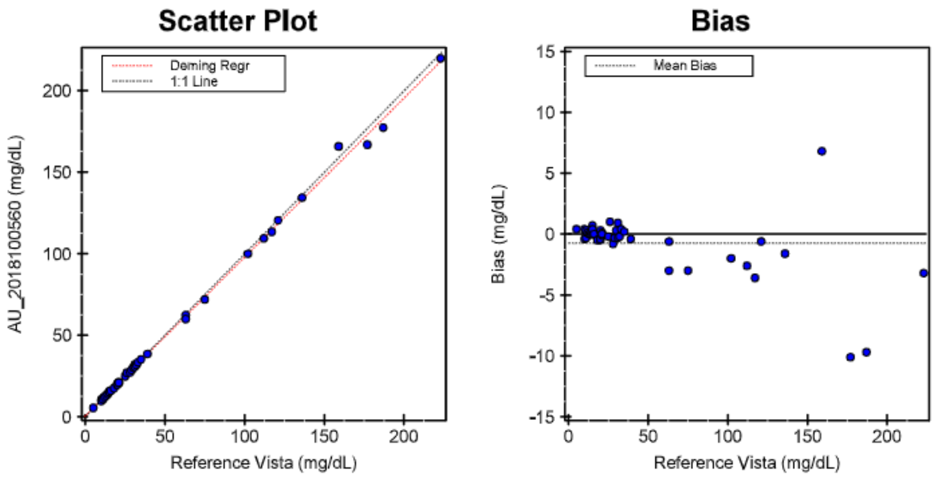


| Slope | Intercept | R | S.E.E. | Mean X | Mean Y | N |
|---|---|---|---|---|---|---|
| 0.976 | 0.48 | 0.9992 | 2.2 | 50.53 | 49.79 | 43 |

**Calcium**

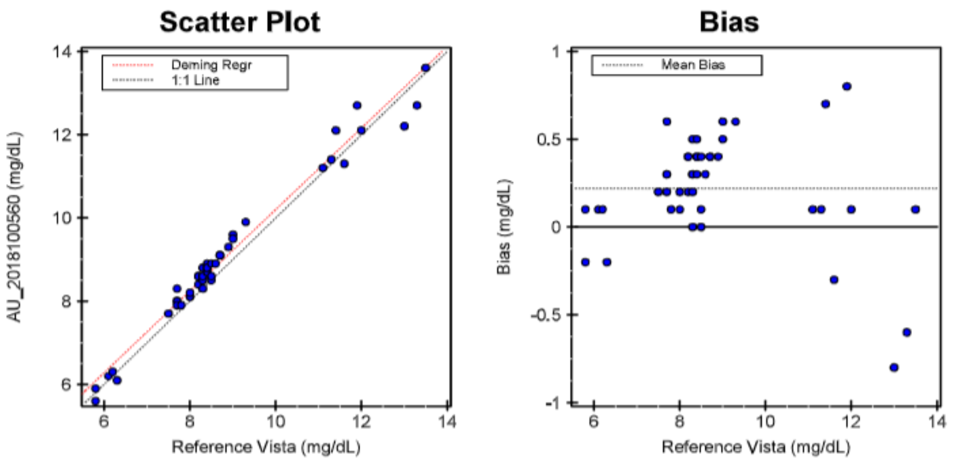


| Slope | Intercept | R | S.E.E. | Mean X | Mean Y | N |
|---|---|---|---|---|---|---|
| 0.98 | 0.4 | 0.9868 | 0.31 | 8.84 | 9.06 | 44 |

**Creatinine**

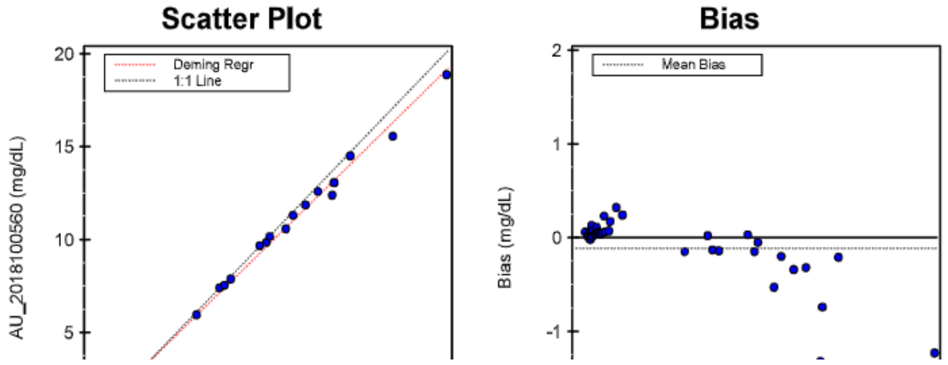

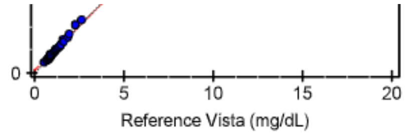


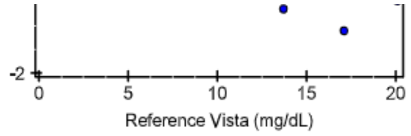


| Slope | Intercept | R | S.E.E. | Mean X | Mean Y | N |
|---|---|---|---|---|---|---|
| 0.944 | 0.165 | 0.9990 | 0.235 | 4.976 | 4.862 | 44 |

**Glucose**

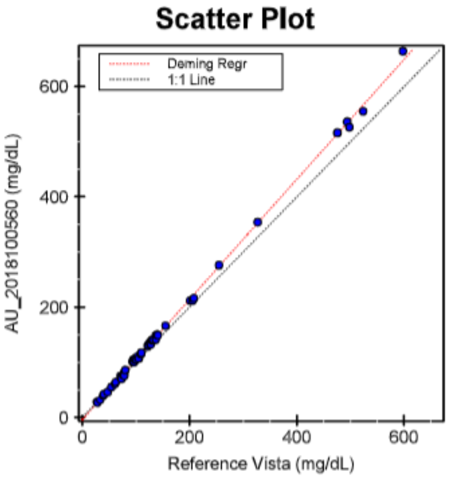


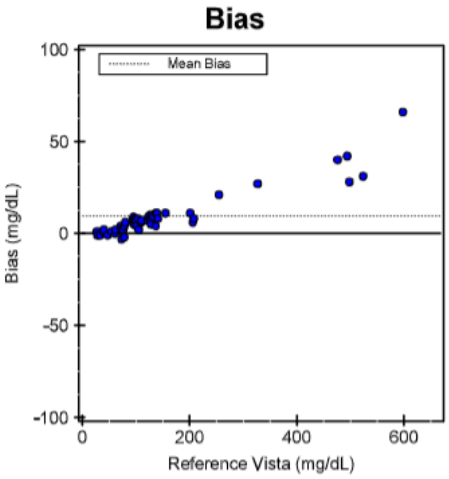


| Slope | Intercept | R | S.E.E. | Mean X | Mean Y | N |
|---|---|---|---|---|---|---|
| 1.088 | -3.6 | 0.9996 | 4.4 | 150.1 | 159.6 | 50 |

**Phosphorus**

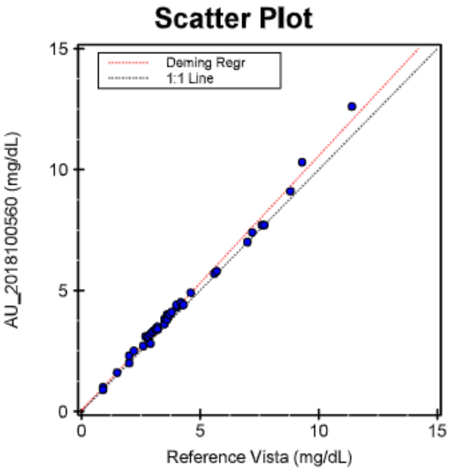


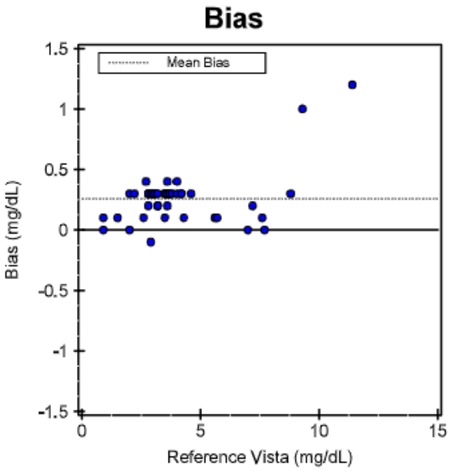


| Slope | Intercept | R | S.E.E. | Mean X | Mean Y | N |
|---|---|---|---|---|---|---|
| 1.053 | 0.04 | 0.9965 | 0.20 | 4.08 | 4.34 | 44 |

**Total Protein**

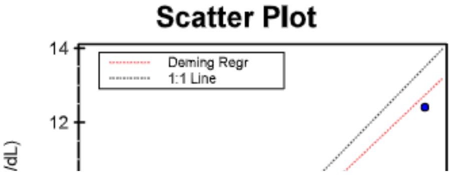


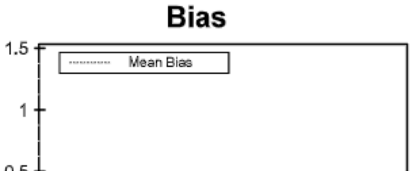

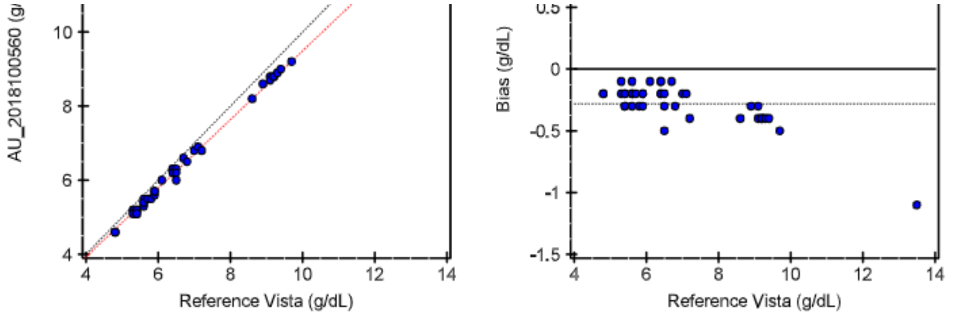


| Slope | Intercept | R | S.E.E. | Mean X | Mean Y | N |
|---|---|---|---|---|---|---|
| 0.926 | 0.23 | 0.9980 | 0.11 | 6.89 | 6.6 | 40 |

**GENERAL CHEMISTRY**

**ALP**

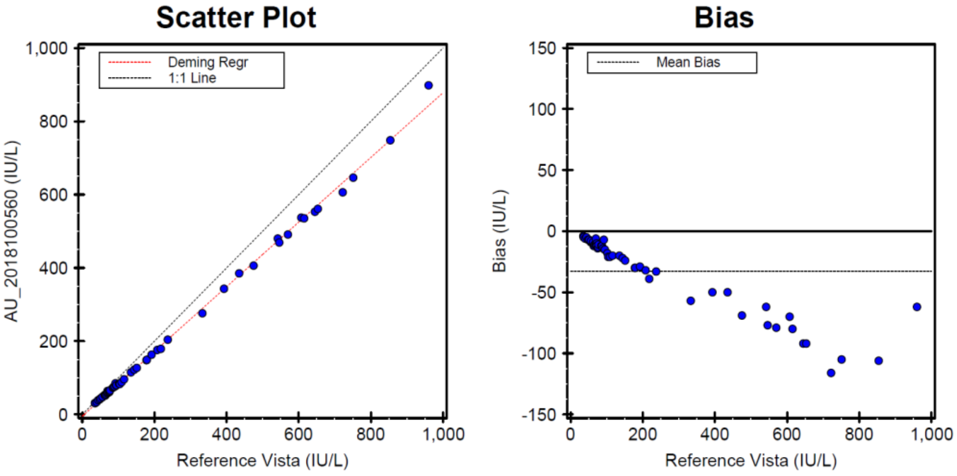


| Slope | Intercept | R | S.E.E. | Mean X | Mean Y | N |
|---|---|---|---|---|---|---|
| 0.883 | -4.6 | 0.999 | 9.9 | 239.3 | 206.6 | 54 |

**ALT**

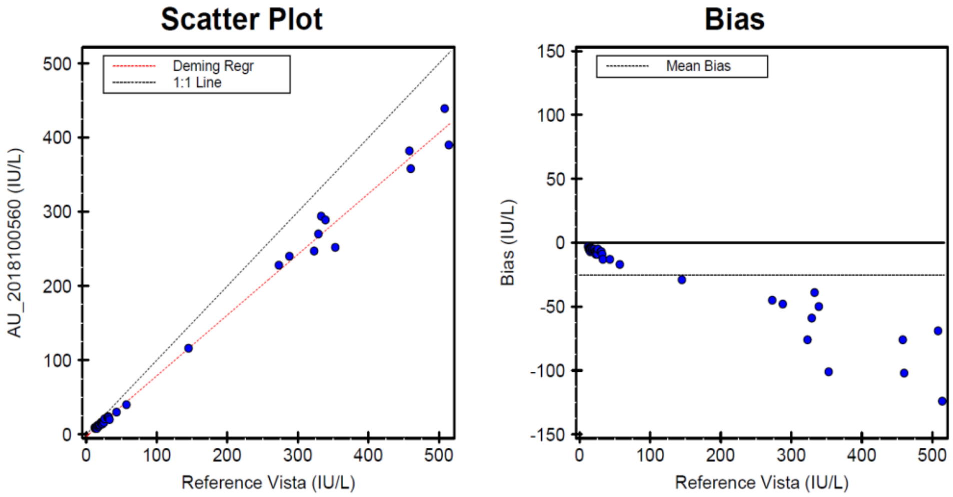


| Slope | Intercept | R | S.E.E. | Mean X | Mean Y | N |
|---|---|---|---|---|---|---|
| 0.818 | -2.8 | 0.9971 | 10.5 | 124.3 | 98.9 | 40 |

**Ammonia**

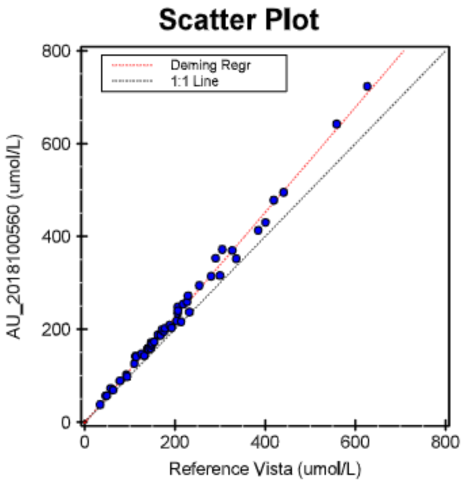


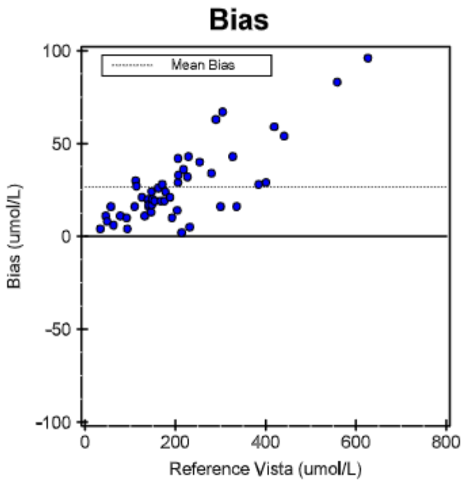


| Slope | Intercept | R | S.E.E. | Mean X | Mean Y | N |
|---|---|---|---|---|---|---|
| 1.131 | -0.5 | 0.9964 | 12.2 | 207.5 | 234.1 | 50 |

**Amylase**

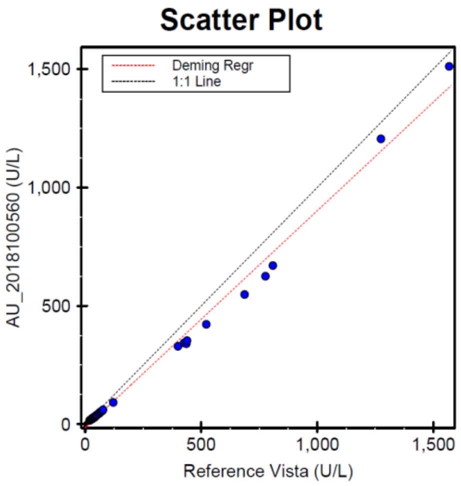


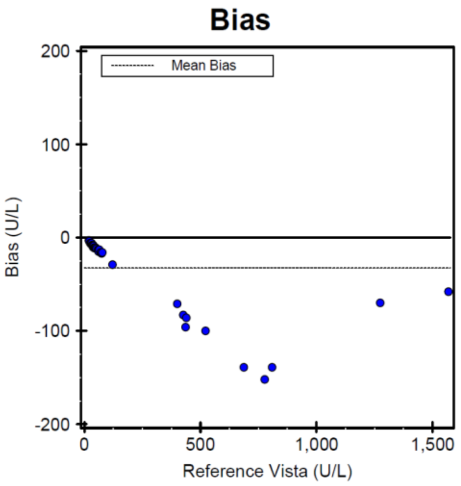


| Slope | Intercept | R | S.E.E. | Mean X | Mean Y | N |
|---|---|---|---|---|---|---|
| 0.916 | -14.5 | 0.9962 | 29 | 217.7 | 185 | 40 |

**AST**

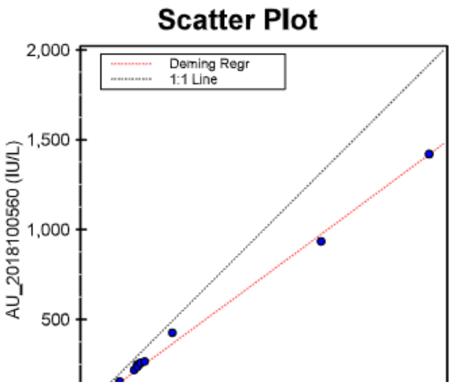


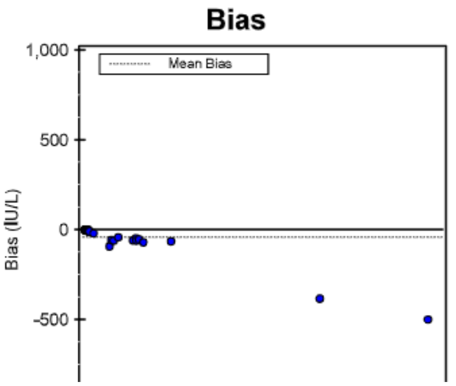

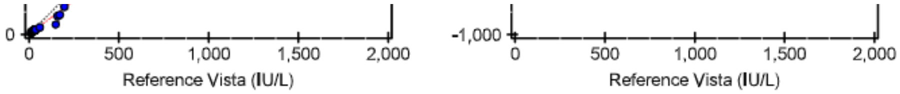


| Slope | Intercept | R | S.E.E. | Mean X | Mean Y | N |
|---|---|---|---|---|---|---|
| 0.738 | 2.1 | 0.9980 | 17.4 | 165.0 | 123.8 | 40 |

**Cholesterol**

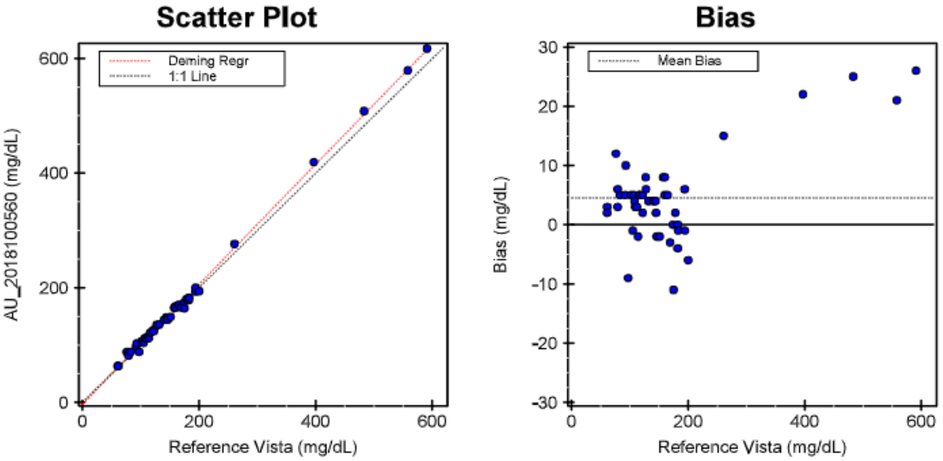


| Slope | Intercept | R | S.E.E. | Mean X | Mean Y | N |
|---|---|---|---|---|---|---|
| 1.046 | -3.2 | 0.9989 | 5.6 | 166.6 | 171.1 | 47 |

**CK**

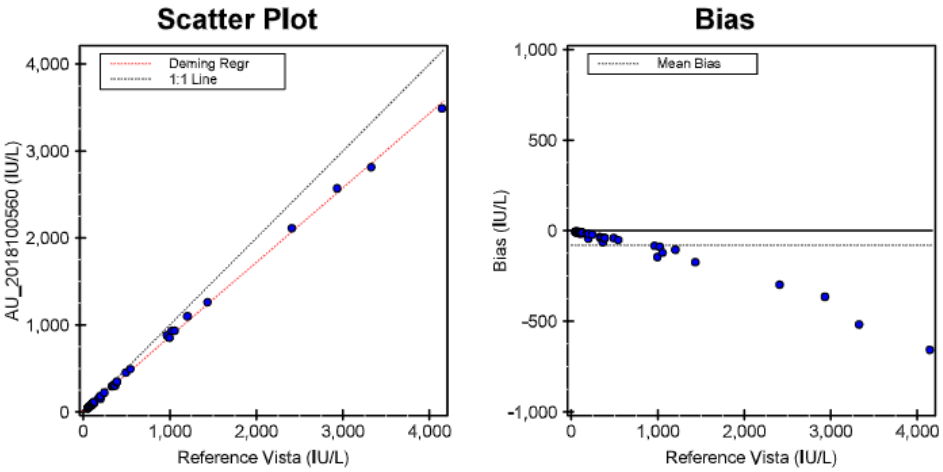


| Slope | Intercept | R | S.E.E. | Mean X | Mean Y | N |
|---|---|---|---|---|---|---|
| 0.855 | 11.2 | 0.9996 | 24.8 | 633.5 | 553.1 | 39 |

**DBILI**

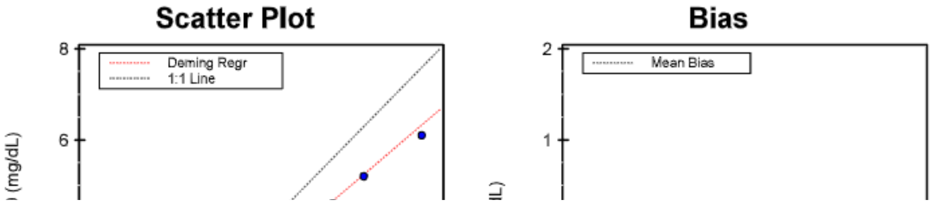

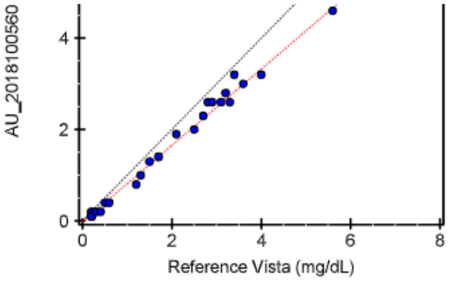


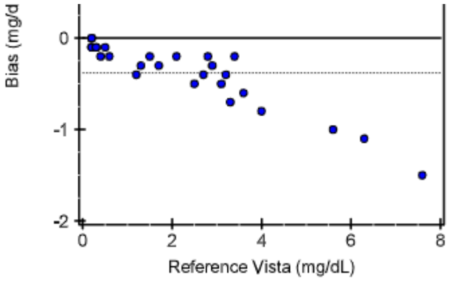


| Slope | Intercept | R | S.E.E. | Mean X | Mean Y | N |
|---|---|---|---|---|---|---|
| 0.835 | -0.01 | 0.9967 | 0.14 | 2.21 | 1.83 | 28 |

**GGT**

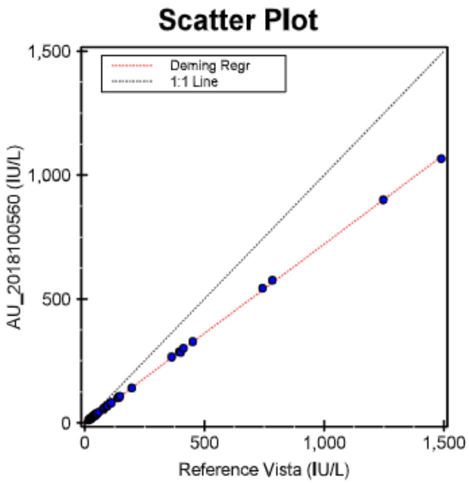


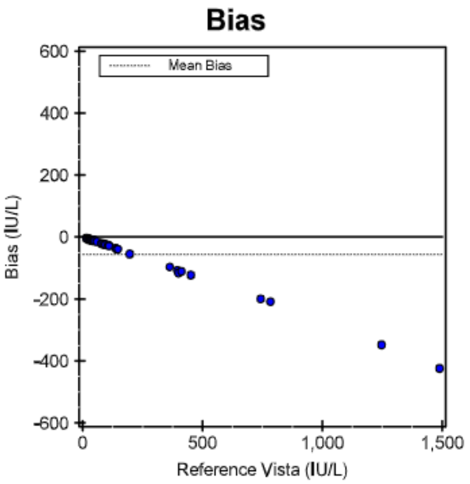


| Slope | Intercept | R | S.E.E. | Mean X | Mean Y | N |
|---|---|---|---|---|---|---|
| 0.720 | 1.2 | 0.9999 | 2.8 | 206.3 | 149.8 | 40 |

**HDL-Chol**

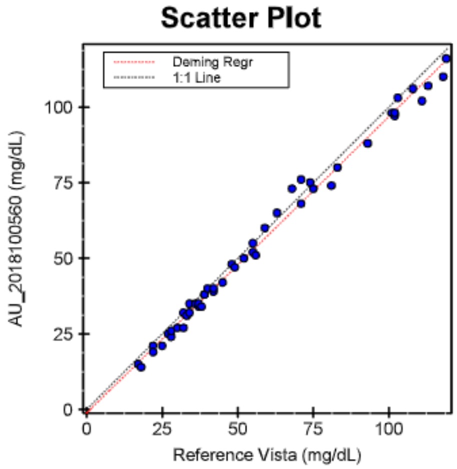


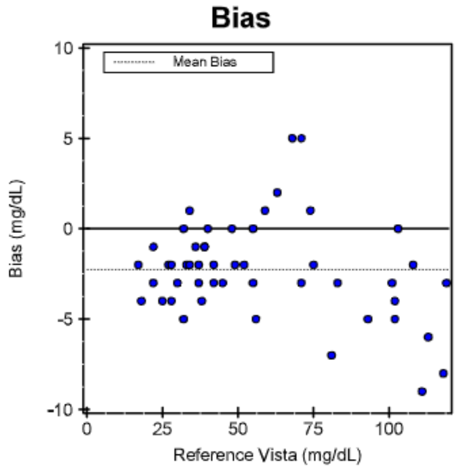


| Slope | Intercept | R | S.E.E. | Mean X | Mean Y | N |
|---|---|---|---|---|---|---|
| 0.980 | -1.1 | 0.9961 | 2.6 | 57.3 | 55.0 | 49 |

**Iron**

Scatter Plot

Bias

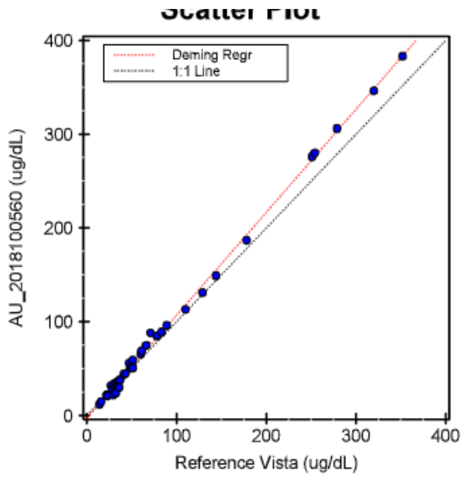


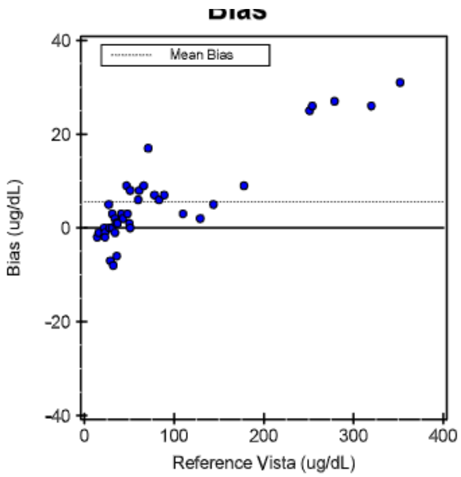


| Slope | Intercept | R | S.E.E. | Mean X | Mean Y | N |
|---|---|---|---|---|---|---|
| 1.097 | -2.5 | 0.9990 | 4.4 | 83.6 | 89.3 | 40 |

**Lactate**

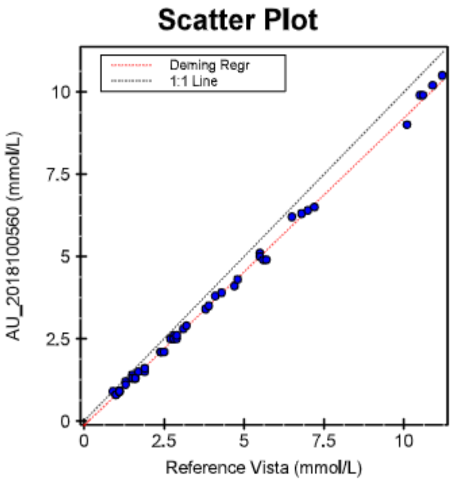


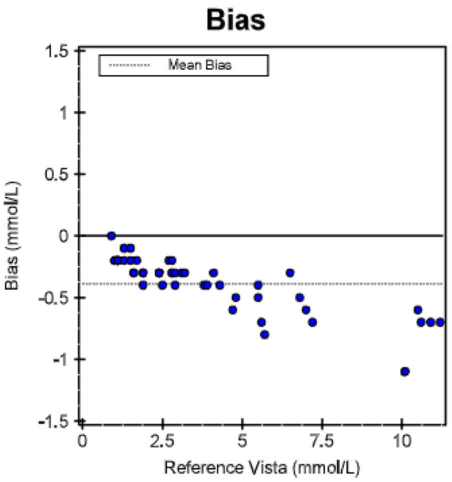


| Slope | Intercept | R | S.E.E. | Mean X | Mean Y | N |
|---|---|---|---|---|---|---|
| 0.934 | -0.13 | 0.9991 | 0.12 | 3.93 | 3.54 | 50 |

**LD**

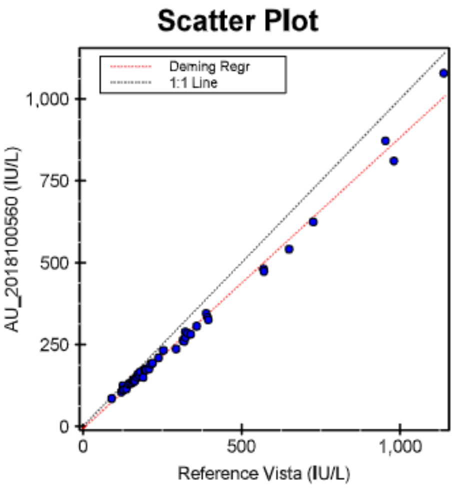


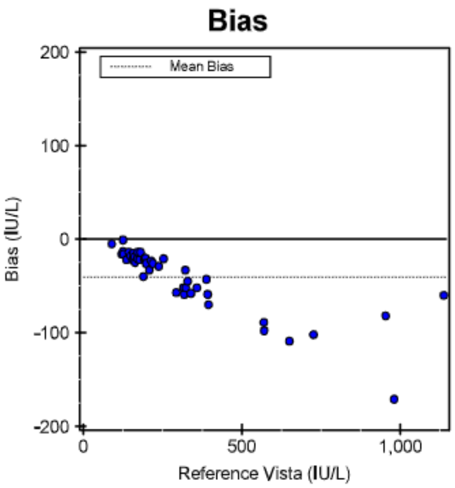

| Slope | Intercept | R | S.E.E. | Mean X | Mean Y | N |
|---|---|---|---|---|---|---|
| 0.888 | -5.9 | 0.9963 | 18.7 | 310.5 | 269.8 | 45 |

**LDL-Chol**

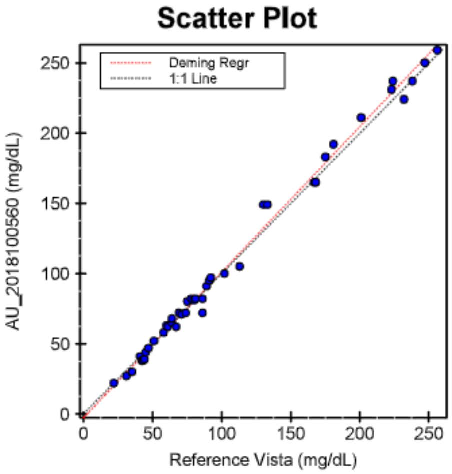


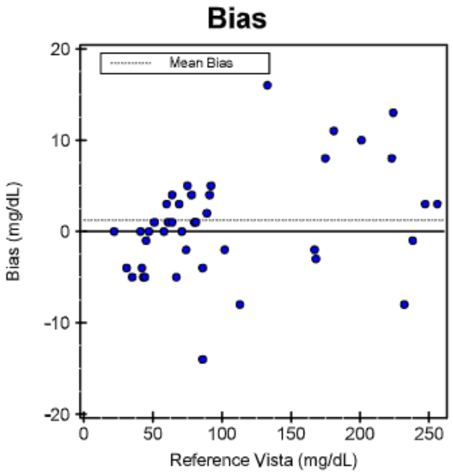


| Slope | Intercept | R | S.E.E. | Mean X | Mean Y | N |
|---|---|---|---|---|---|---|
| 1.036 | -2.6 | 0.9964 | 6.1 | 105.5 | 106.7 | 43 |

**Lipase**

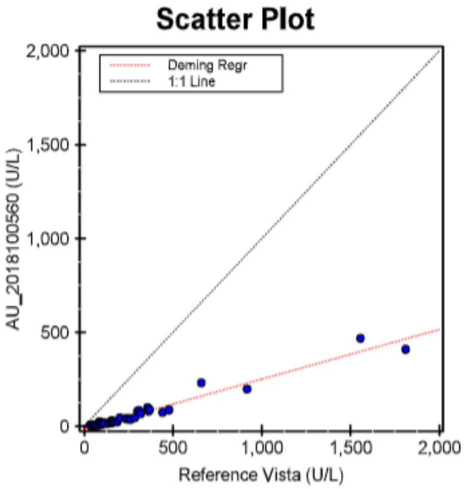


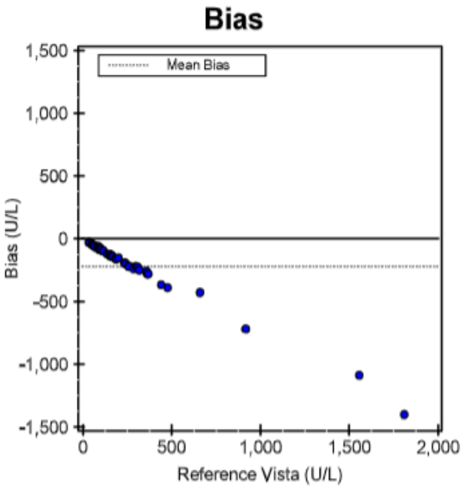


| Slope | Intercept | R | S.E.E. | Mean X | Mean Y | N |
|---|---|---|---|---|---|---|
| 0.265 | -13.7 | 0.9765 | 21.2 | 280.0 | 60.4 | 43 |

**Magnesium**

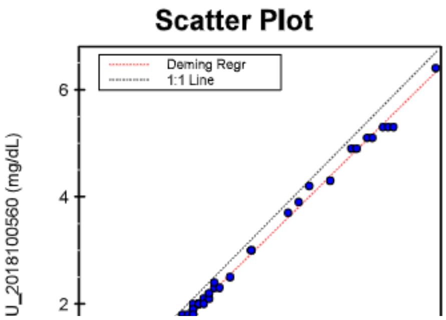


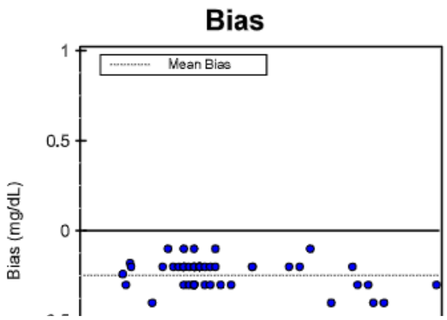

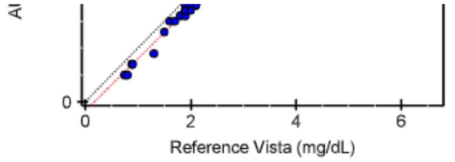


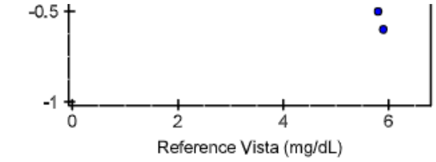


| Slope | Intercept | R | S.E.E. | Mean X | Mean Y | N |
|---|---|---|---|---|---|---|
| 0.969 | -0.16 | 0.9982 | 0.086 | 2.754 | 2.508 | 52 |

**TBILI**

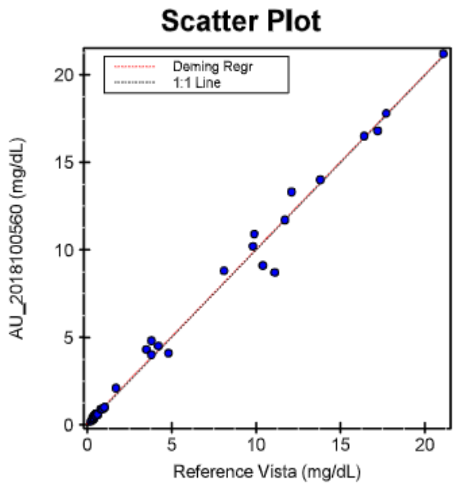


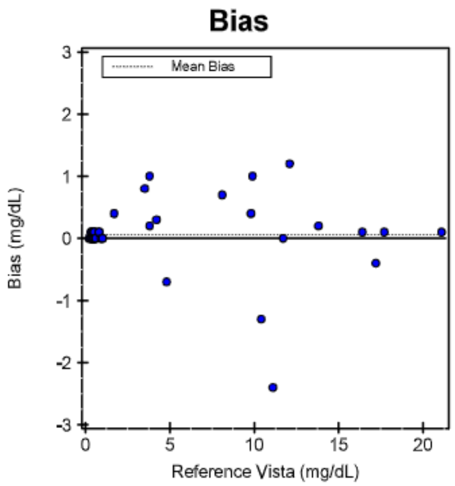


| Slope | Intercept | R | S.E.E. | Mean X | Mean Y | N |
|---|---|---|---|---|---|---|
| 0.998 | 0.07 | 0.9958 | 0.55 | 4.25 | 4.31 | 46 |

**Triglyceride**

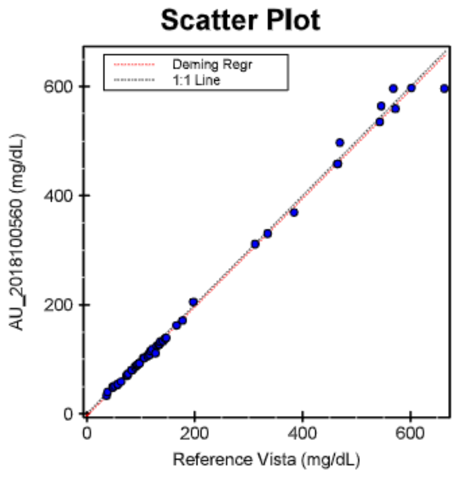


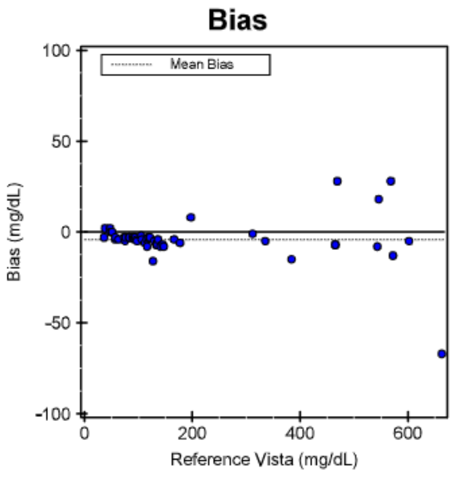


| Slope | Intercept | R | S.E.E. | Mean X | Mean Y | N |
|---|---|---|---|---|---|---|
| 0.995 | -3.1 | 0.9977 | 12.1 | 194.4 | 190.4 | 51 |

**Uric Acid**

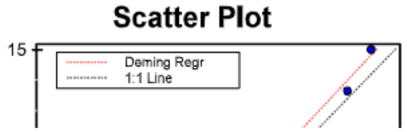


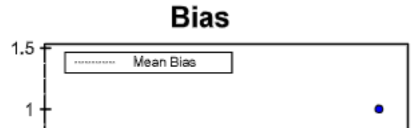

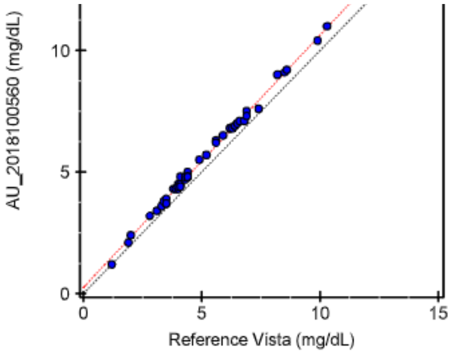


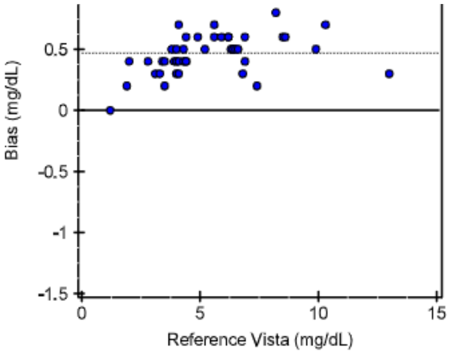


| Slope | Intercept | R | S.E.E. | Mean X | Mean Y | N |
|---|---|---|---|---|---|---|
| 1.038 | 0.26 | 0.9986 | 0.15 | 5.57 | 6.04 | 45 |

**PROTEINS**

**Complement C3**

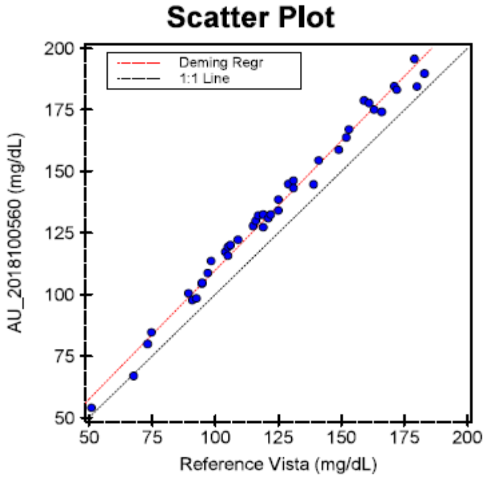


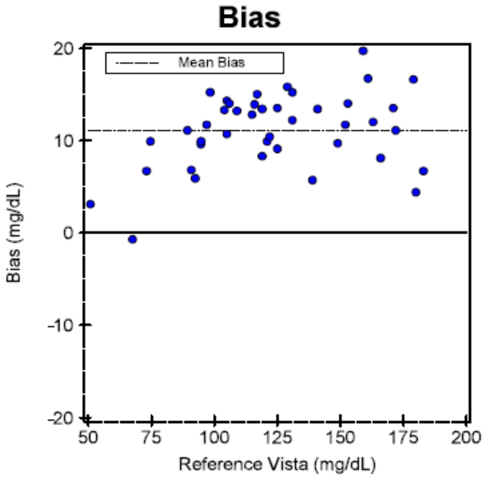


| Slope | Intercept | R | S.E.E. | Mean X | Mean Y | N |
|---|---|---|---|---|---|---|
| 1.048 | 5.22 | 0.9939 | 3.86 | 123.6 | 134.7 | 42 |

**Complement C4**

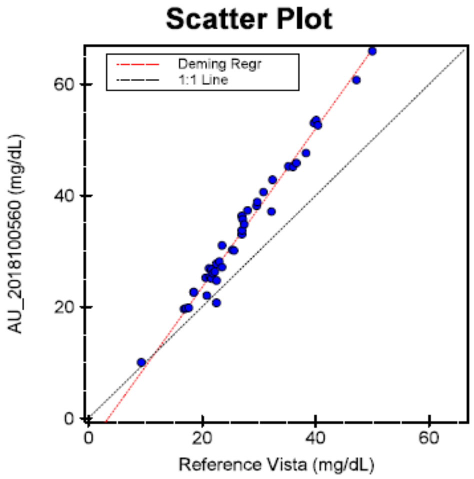


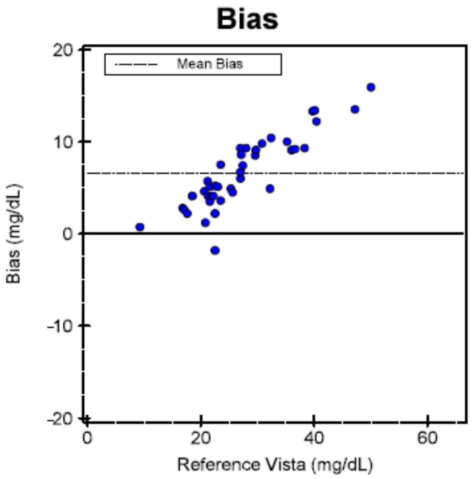

| Slope | Intercept | R | S.E.E. | Mean X | Mean Y | N |
|---|---|---|---|---|---|---|
| 1.42 | -4.9 | 0.9892 | 1.8 | 27.1 | 33.7 | 42 |

**CRP**

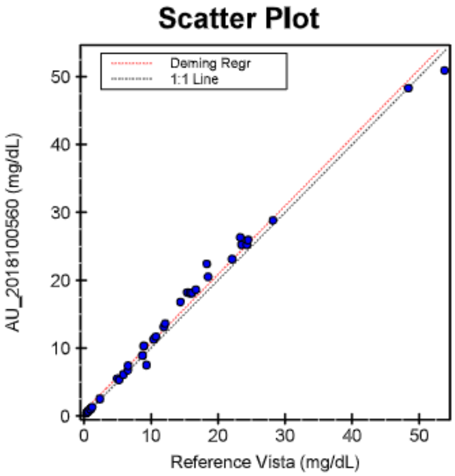


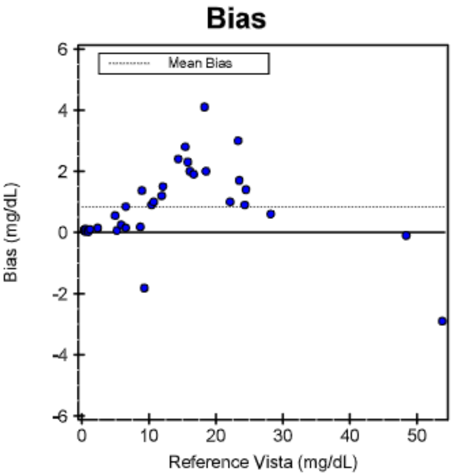


| Slope | Intercept | R | S.E.E. | Mean X | Mean Y | N |
|---|---|---|---|---|---|---|
| 1.006 | 0.7591 | 0.9947 | 1.3149 | 13.1164 | 13.95 | 36 |

**CRP-hs**

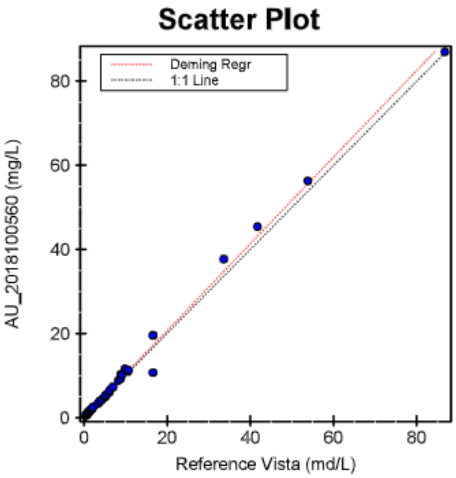


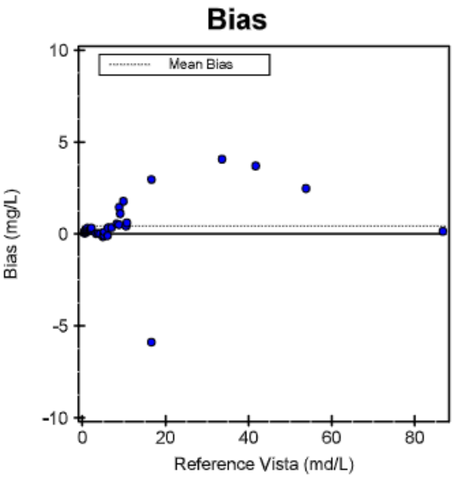


| Slope | Intercept | R | S.E.E. | Mean X | Mean Y | N |
|---|---|---|---|---|---|---|
| 1.029 | 0.1573 | 0.9969 | 1.3712 | 9.4314 | 9.8639 | 41 |

**Ferritin**

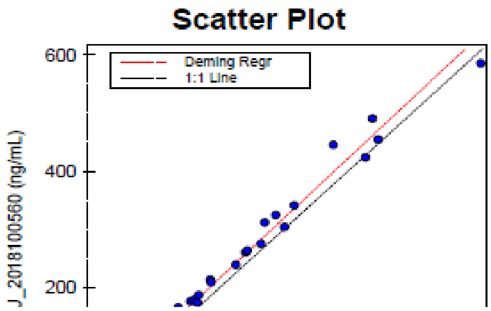


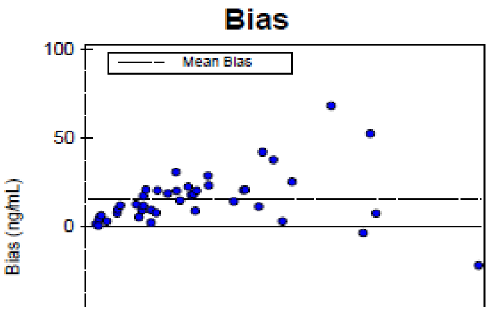

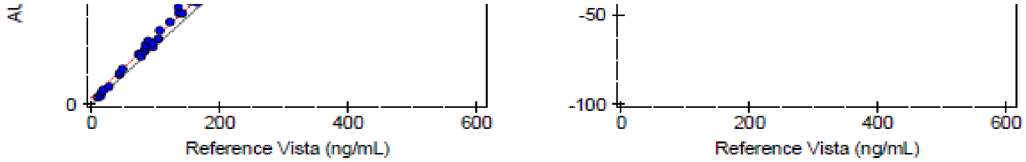


| Slope | Intercept | R | S.E.E. | Mean X | Mean Y | N |
|---|---|---|---|---|---|---|
| 1.028 | 10.81 | 0.9944 | 15.09 | 172.9 | 188.5 | 42 |

**IgA**

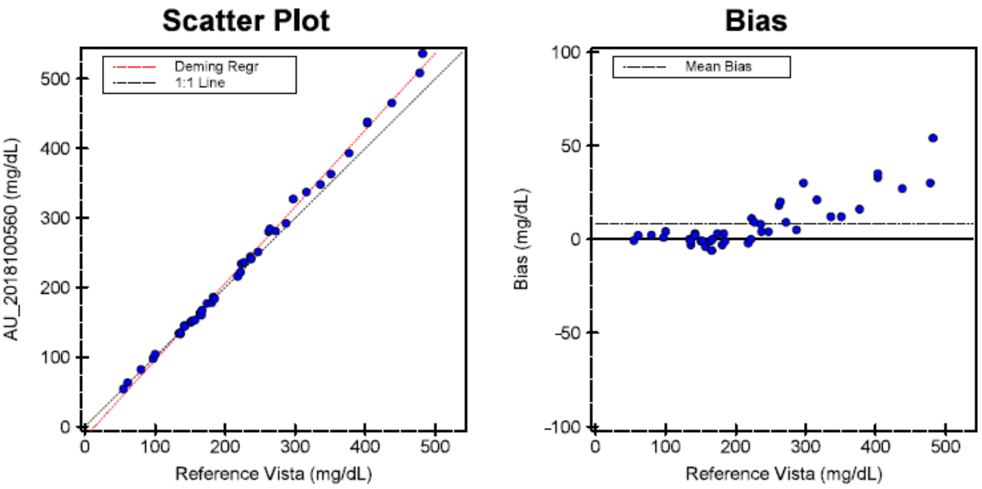


| Slope | Intercept | R | S.E.E. | Mean X | Mean Y | N |
|---|---|---|---|---|---|---|
| 1.10 | -14.8 | 0.9984 | 7.01 | 225.9 | 234.4 | 42 |

**IgG**

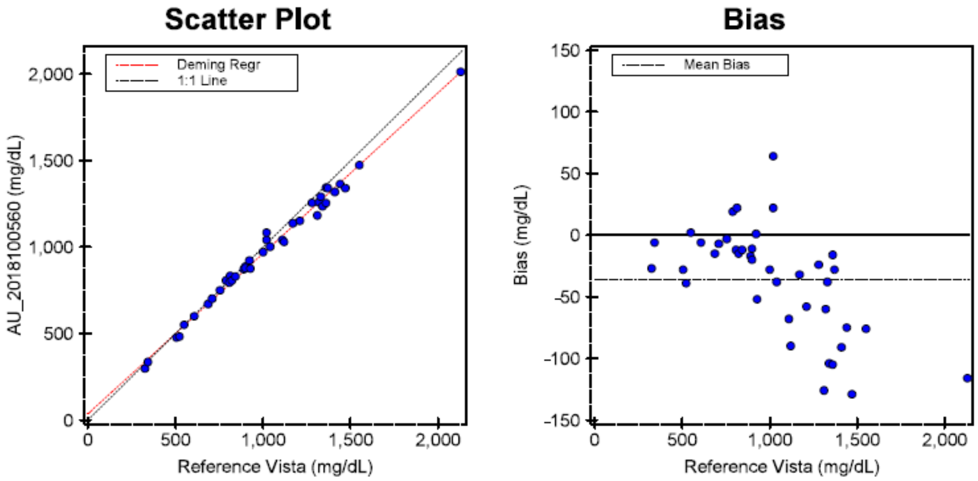


| Slope | Intercept | R | S.E.E. | Mean X | Mean Y | N |
|---|---|---|---|---|---|---|
| 0.929 | 36.8 | 0.9952 | 34.1 | 1024.2 | 988.1 | 40 |

**IgM**

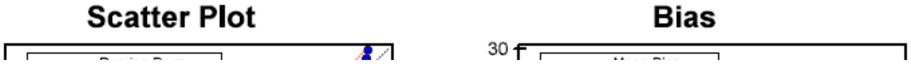

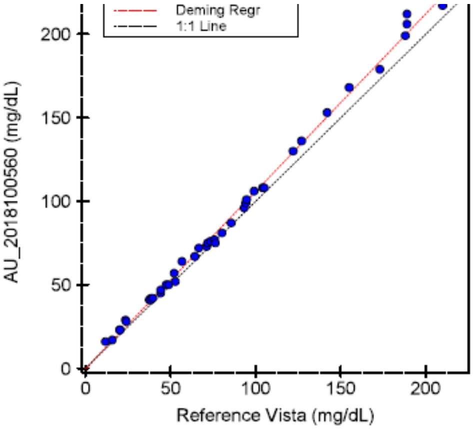


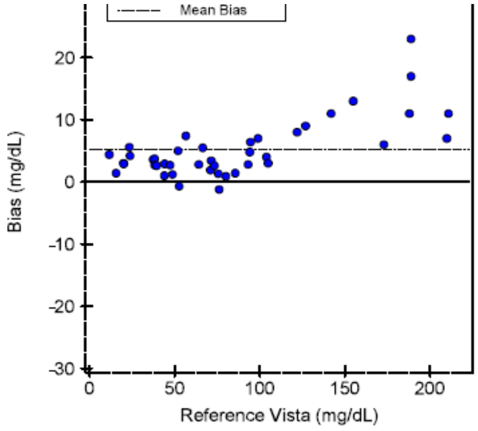


| Slope | Intercept | R | S.E.E. | Mean X | Mean Y | N |
|---|---|---|---|---|---|---|
| 1.06 | -0.1 | 0.9985 | 3.34 | 85.1 | 90.2 | 42 |

**Prealbumin**

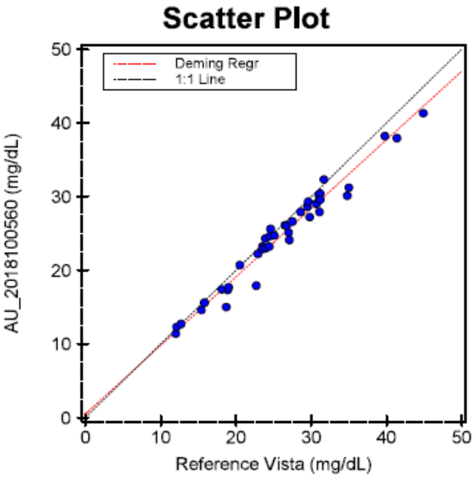


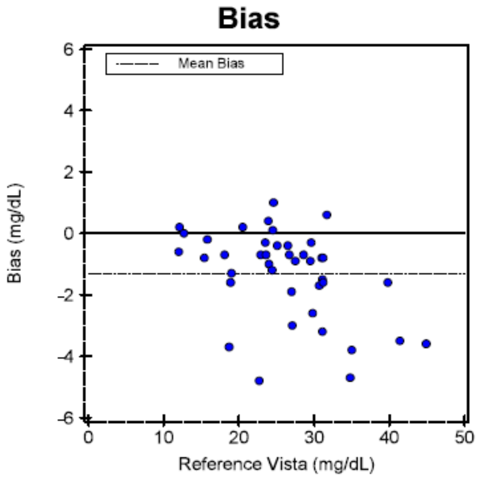


| Slope | Intercept | R | S.E.E. | Mean X | Mean Y | N |
|---|---|---|---|---|---|---|
| 0.93 | 0.54 | 0.9828 | 1.3 | 26.1 | 24.8 | 41 |

**RF**

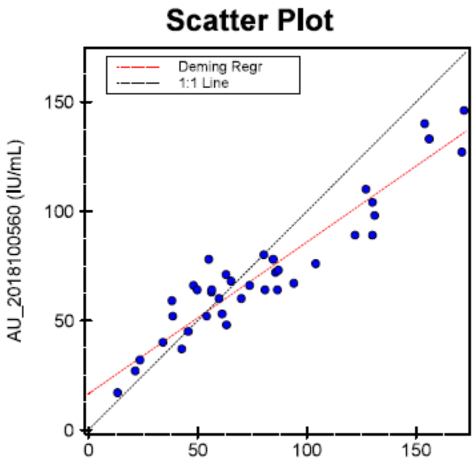


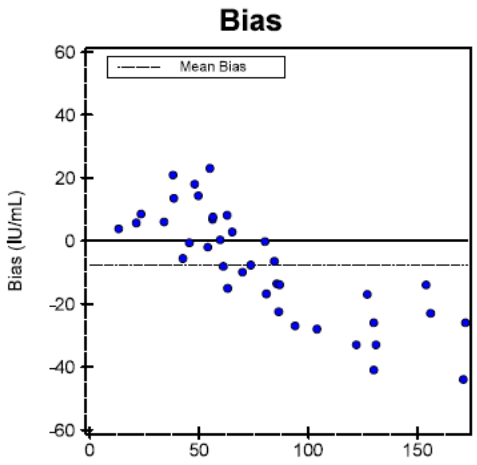

Reference Vista (IU/mL) Reference Vista (IU/mL)

| Slope | Intercept | R | S.E.E. | Mean X | Mean Y | N |
|---|---|---|---|---|---|---|
| 0.70 | 16.6 | 0.9413 | 10.3 | 79.7 | 71.9 | 38 |

**Transferrin**

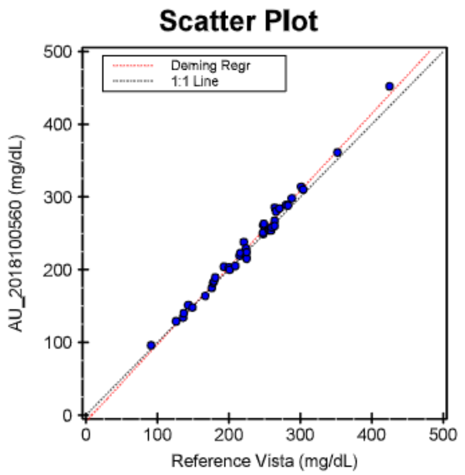


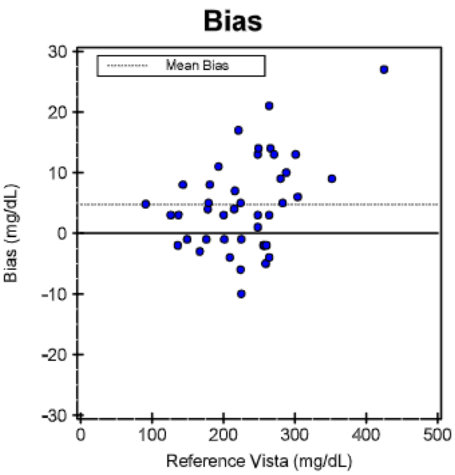


| Slope | Intercept | R | S.E.E. | Mean X | Mean Y | N |
|---|---|---|---|---|---|---|
| 1.057 | -8.30 | 0.9946 | 7.04 | 228.65 | 233.4 | 42 |

**URINES**

**U-Albumin**

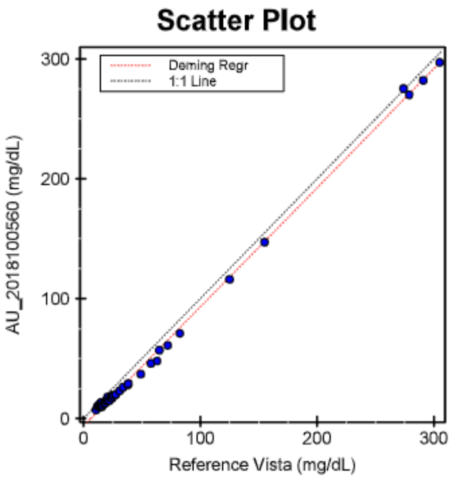


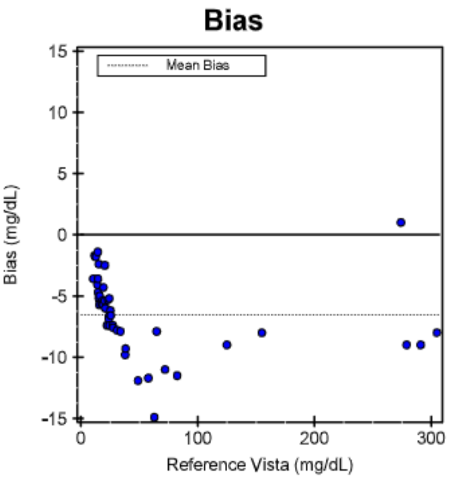


| Slope | Intercept | R | S.E.E. | Mean X | Mean Y | N |
|---|---|---|---|---|---|---|
| 0.993 | -6.14 | 0.9992 | 3.13 | 56.76 | 50.25 | 44 |

**U-BUN**

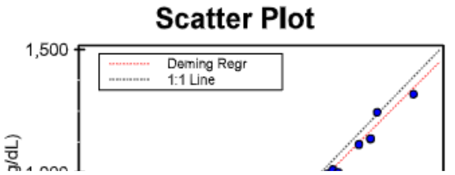


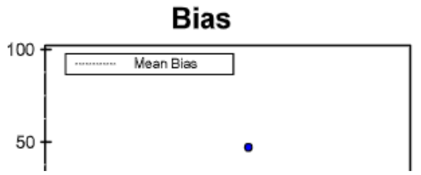

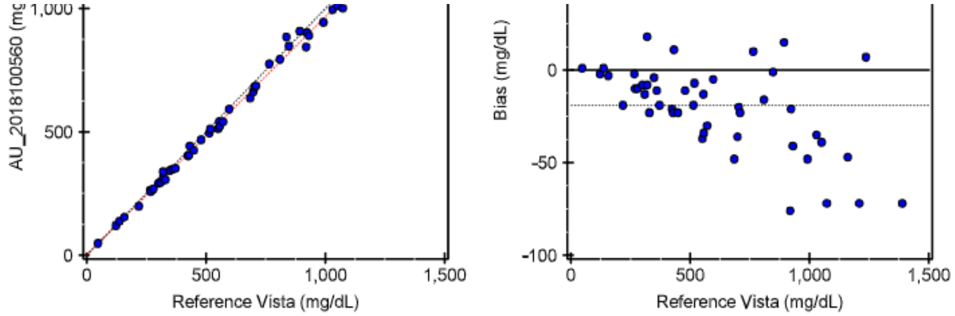


| Slope | Intercept | R | S.E.E. | Mean X | Mean Y | N |
|---|---|---|---|---|---|---|
| 0.965 | 2.3 | 0.9978 | 21.6 | 606 | 587 | 48 |

**U-Calcium**

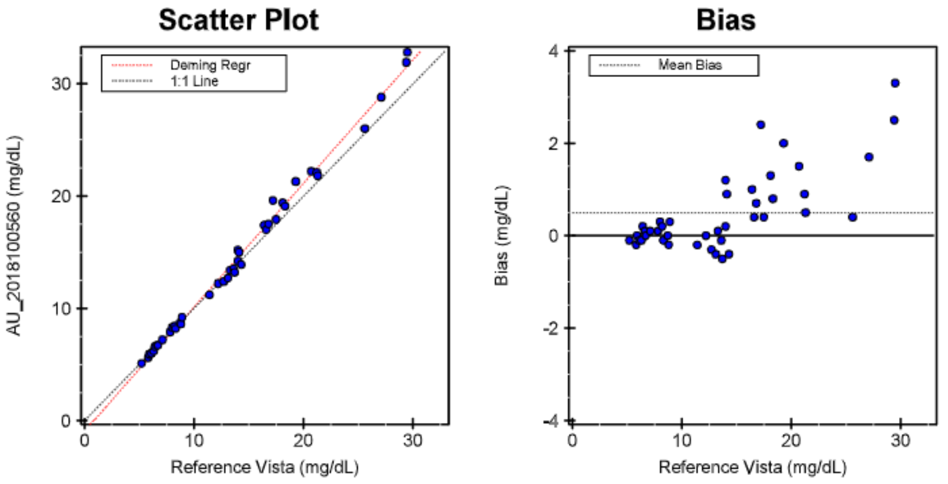


| Slope | Intercept | R | S.E.E. | Mean X | Mean Y | N |
|---|---|---|---|---|---|---|
| 1.100 | -0.88 | 0.9969 | 0.58 | 13.72 | 14.21 | 42 |

**U-Chloride**

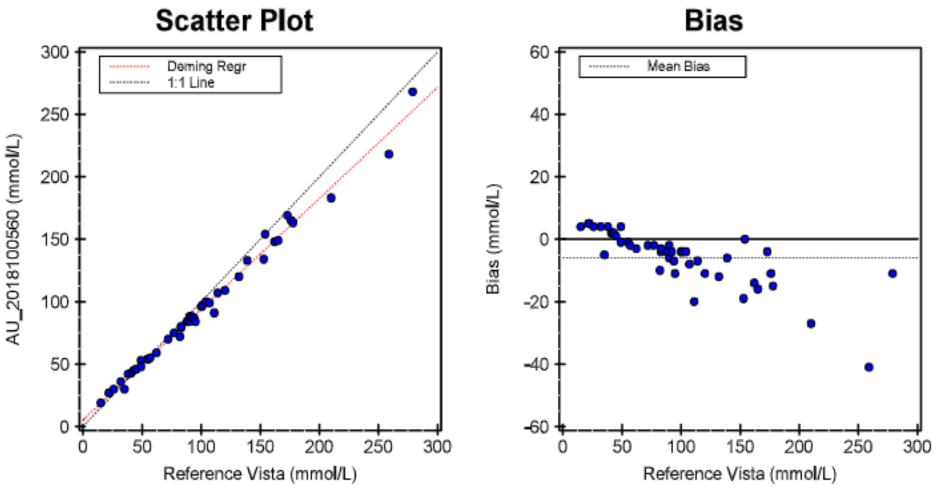


| Slope | Intercept | R | S.E.E. | Mean X | Mean Y | N |
|---|---|---|---|---|---|---|
| 0.887 | 5.3 | 0.9949 | 5.5 | 99.4 | 93.5 | 45 |

**U-Creatinine**

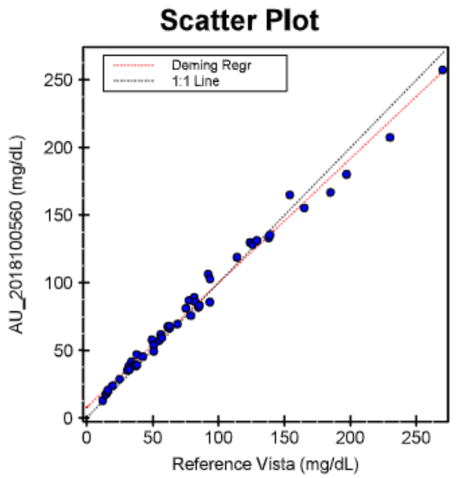


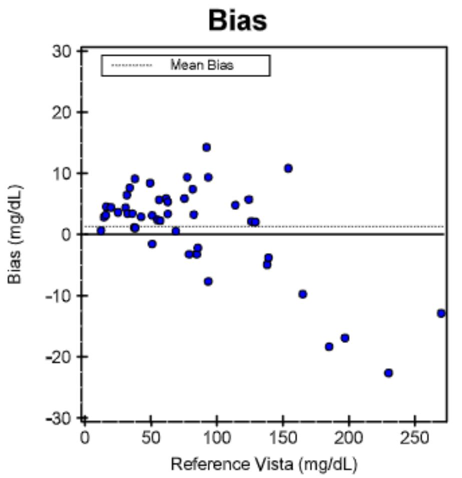


| Slope | Intercept | R | S.E.E. | Mean X | Mean Y | N |
|---|---|---|---|---|---|---|
| 0.920 | 7.834 | 0.9945 | 5.72 | 81.155 | 82.492 | 47 |

**U-Glucose**

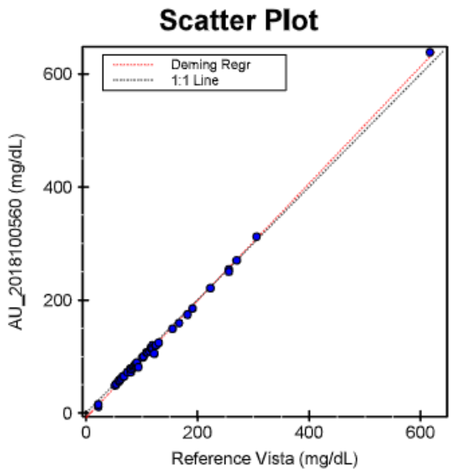


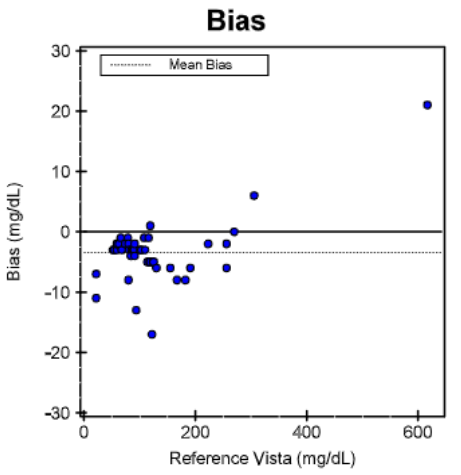


| Slope | Intercept | R | S.E.E. | Mean X | Mean Y | N |
|---|---|---|---|---|---|---|
| 1.034 | -7.7 | 0.9992 | 4.1 | 126.1 | 122.7 | 45 |

**U-Phosphorus**

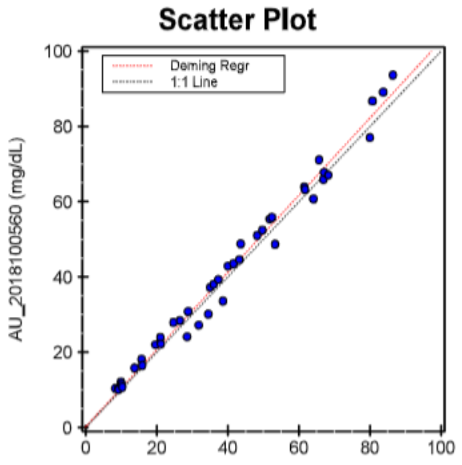


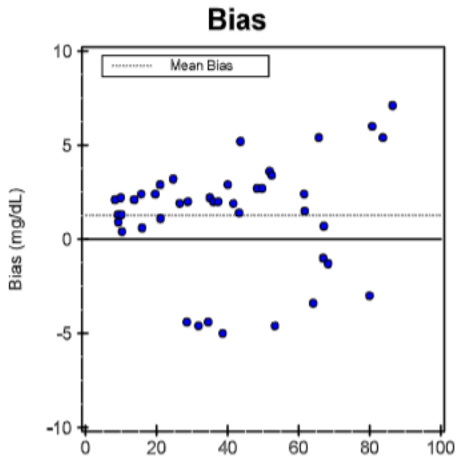

Reference Vista (mg/dL) Reference Vista (mg/dL)

| Slope | Intercept | R | S.E.E. | Mean X | Mean Y | N |
|---|---|---|---|---|---|---|
| 1.025 | 0.28 | 0.9921 | 3.000 | 40.37 | 41.65 | 42 |

**U-Potassium**

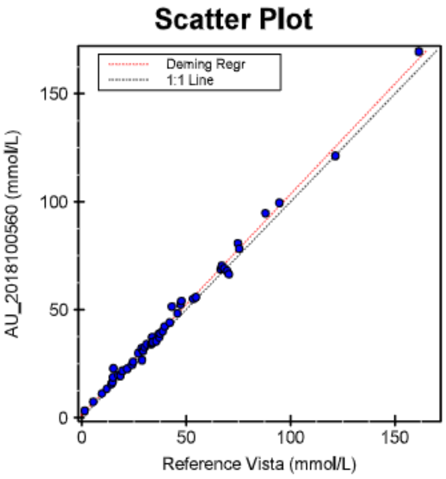


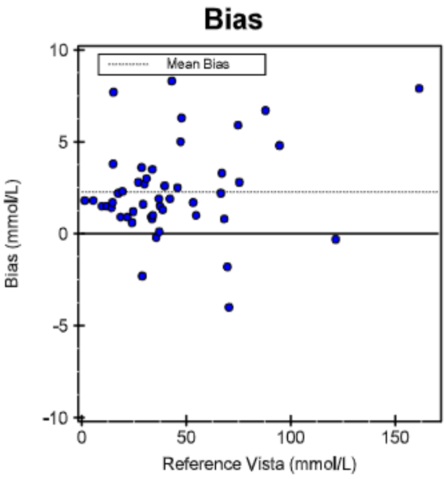


| Slope | Intercept | R | S.E.E. | Mean X | Mean Y | N |
|---|---|---|---|---|---|---|
| 1.021 | 1.37 | 0.9970 | 2.43 | 42.47 | 44.74 | 48 |

**U-Sodium**

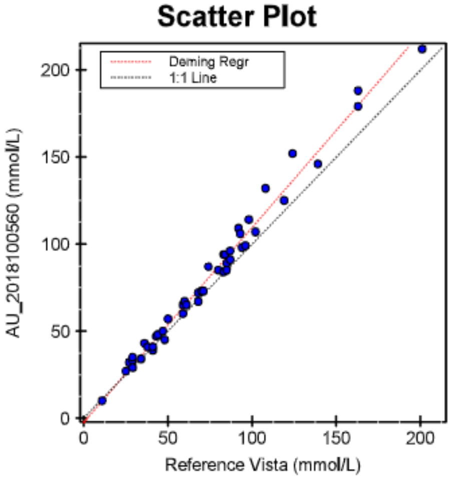


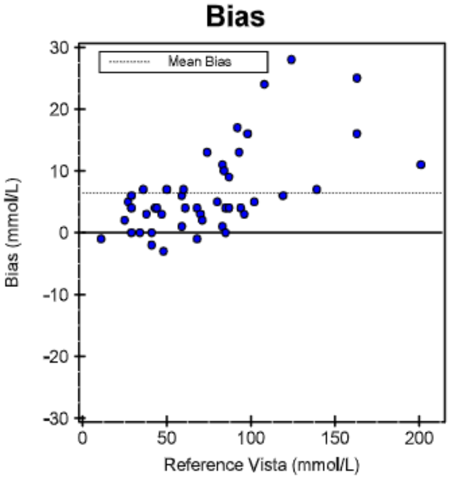


| Slope | Intercept | R | S.E.E. | Mean X | Mean Y | N |
|---|---|---|---|---|---|---|
| 1.118 | -2.3 | 0.9924 | 5.6 | 74.5 | 81.0 | 46 |

**U-Total Protein**

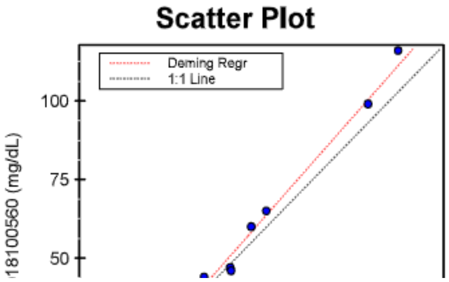


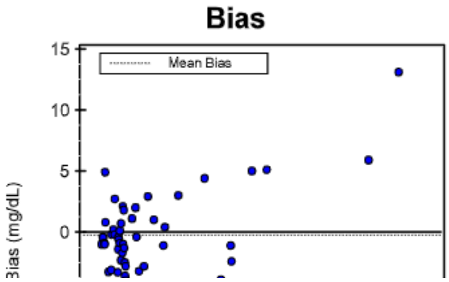

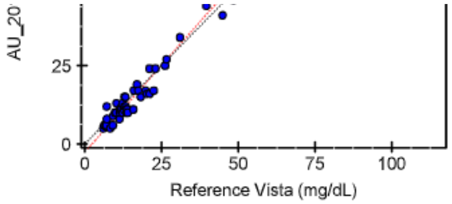


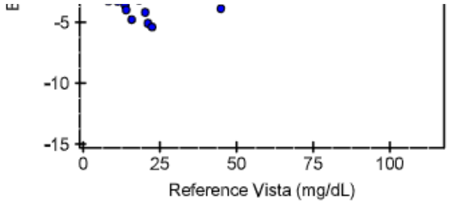


| Slope | Intercept | R | S.E.E. | Mean X | Mean Y | N |
|---|---|---|---|---|---|---|
| 1.106 | -2.517 | 0.9926 | 2.729 | 21.259 | 21.000 | 52 |

## CSF

### CSF-Glucose

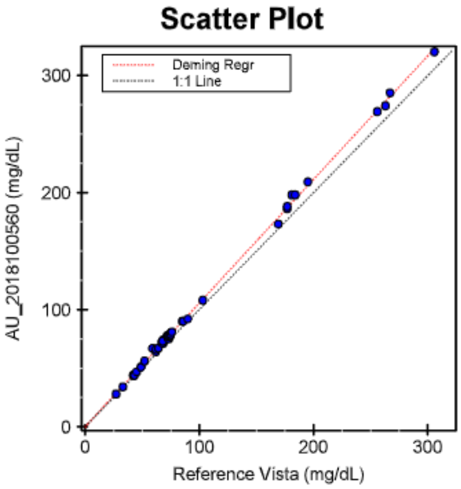


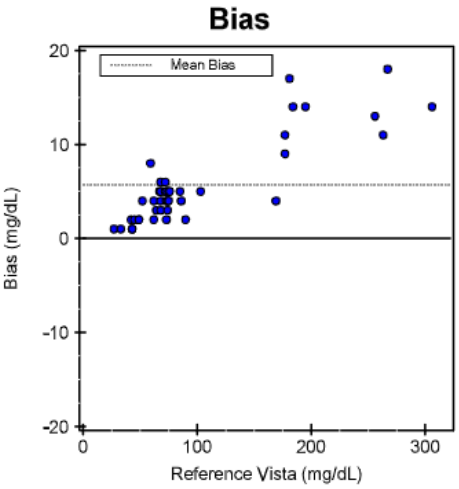


| Slope | Intercept | R | S.E.E. | Mean X | Mean Y | N |
|---|---|---|---|---|---|---|
| 1.056 | 0.1 | 0.9996 | 2.2 | 100.3 | 106.0 | 43 |

### CSF-Total Protein

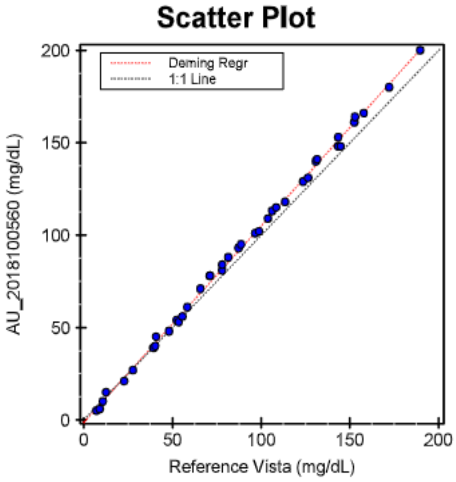


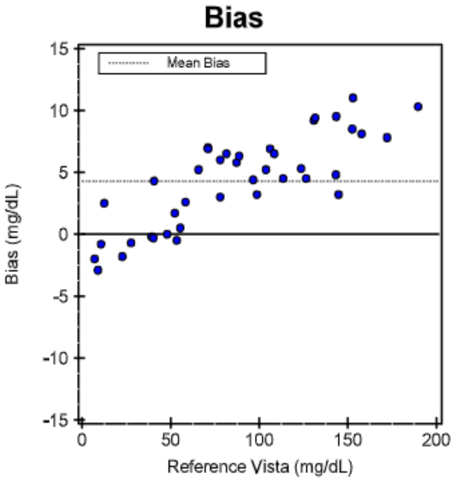


| Slope | Intercept | R | S.E.E. | Mean X | Mean Y | N |
|---|---|---|---|---|---|---|
| 1.063 | -1.25 | 0.9992 | 2.11 | 87.39 | 91.67 | 40 |
